# Design and applicability of DNA arrays and DNA barcodes in biodiversity monitoring

**DOI:** 10.1186/1741-7007-5-24

**Published:** 2007-06-13

**Authors:** Mehrdad Hajibabaei, Gregory AC Singer, Elizabeth L Clare, Paul DN Hebert

**Affiliations:** 1 Biodiversity Institute of Ontario, Department of Integrative Biology, University of Guelph, Guelph, Ontario, N1G 2W1, Canada; 2 Human Cancer Genetics Program, The Ohio State University, Columbus, OH, 43210, USA

## Abstract

**Background:**

The rapid and accurate identification of species is a critical component of large-scale biodiversity monitoring programs. DNA arrays (micro and macro) and DNA barcodes are two molecular approaches that have recently garnered much attention. Here, we compare these two platforms for identification of an important group, the mammals.

**Results:**

Our analyses, based on the two commonly used mitochondrial genes cytochrome *c *oxidase I (the standard DNA barcode for animal species) and cytochrome b (a common species-level marker), suggest that both arrays and barcodes are capable of discriminating mammalian species with high accuracy. We used three different datasets of mammalian species, comprising different sampling strategies. For DNA arrays we designed three probes for each species to address intraspecific variation. As for DNA barcoding, our analyses show that both cytochrome *c *oxidase I and cytochrome b genes, and even smaller fragments of them (mini-barcodes) can successfully discriminate species in a wide variety of specimens.

**Conclusion:**

This study showed that DNA arrays and DNA barcodes are valuable molecular methods for biodiversity monitoring programs. Both approaches were capable of discriminating among mammalian species in our test assemblages. However, because designing DNA arrays require advance knowledge of target sequences, the use of this approach could be limited in large scale monitoring programs where unknown haplotypes might be encountered. DNA barcodes, by contrast, are sequencing-based and therefore could provide more flexibility in large-scale studies.

## Background

Species identification is essential for large-scale biodiversity monitoring and conservation [1]. Several molecular methods have been employed for biodiversity studies, but traditional methods such as allozyme analysis are usually labor-intensive and irreproducible. Because of advances in DNA-based technologies, approaches such as DNA arrays and DNA barcoding have recently gained attention. Both of these methods are based on comparative DNA sequence analysis, but they have significant differences.

Micro- and macro-arrays rely on the hybridization of short (i.e. 25 base) specific nucleotide probes to DNA from the target organism and subsequent detection of the hybridization signal. Although array-based technologies have been widely used in gene expression studies, their use in biodiversity research has been less rigorous, mainly targeting pathogenic microorganisms [2] and arrays of environmental samples [3]. Pfunder et al [4], however, have advocated an array-based method for the identification of voles and shrews for biodiversity monitoring. Although this study focuses on a limited number of species, the authors have predicted that such an approach can be used for the development of a so called 'Mammalia Chip', in the case of mammalian species, or even a 'Biodiversity Chip' for monitoring key species of different taxa from bacteria to mammals [4].

Species identification by DNA barcoding is based on sequencing a short standardized genomic region of the target specimen and comparing this information to a sequence library from known species [5]. The proposed standard barcode sequence for animal species is a 650-bp fragment of the mitochondrial gene cytochrome *c *oxidase I (COI, *cox1*). This DNA barcode has successfully been used for the identification of species in various vertebrate and invertebrate groups from birds to Lepidoptera [6-8], and in different geographical settings from the arctic to the tropics [6,9]. Additionally, smaller fragments (i.e. 100 bases) of the standard COI barcode – 'mini-barcodes' – have been shown to be effective for species identification in specimens whose DNA is degraded or potentially in other situations where obtaining a full-length barcode is not feasible [10]. Barcoding is now being extended to other groups such as fungi, plants and protists, and the Barcode of Life Initiative has gained international momentum by the establishment of the Consortium for the Barcode of Life (CBOL), which plans to assemble DNA barcode libraries for all fish and birds [11].

Here, we compare the design and applicability of both array-based and barcoding platforms for specimen identification in mammalian species. We have chosen mammals because they constitute an important target for biodiversity studies and include many endangered species. However, mammalian species have not been broadly targeted for developing array-based or barcoding identification systems previously. A rapid identification method will aid in the tracking of illegal trafficking of mammalian species and their tissues. We have selected two mitochondrial loci for our analysis: COI – the proposed standard animal DNA barcode – and cytochrome b (*cytb*), which is commonly used as a species-level marker and particularly so in mammalian biosystematics [4,12]. We used both of these genes to test the possibility of designing a Mammalia Chip. We also used these sequences and various size fragments of them to test the feasibility of DNA barcoding analysis for mammalian species. We targeted three datasets of mammalian species for these analyses: 121 species across the taxonomy of mammals (mammalian dataset), a dense sampling of 87 species of neotropical bats (bat dataset), and a wide geographical sampling of a single genetically diverse bat species (*Sturnira lilium *dataset).

## Results

### Array-based analysis

**Figure 1:**
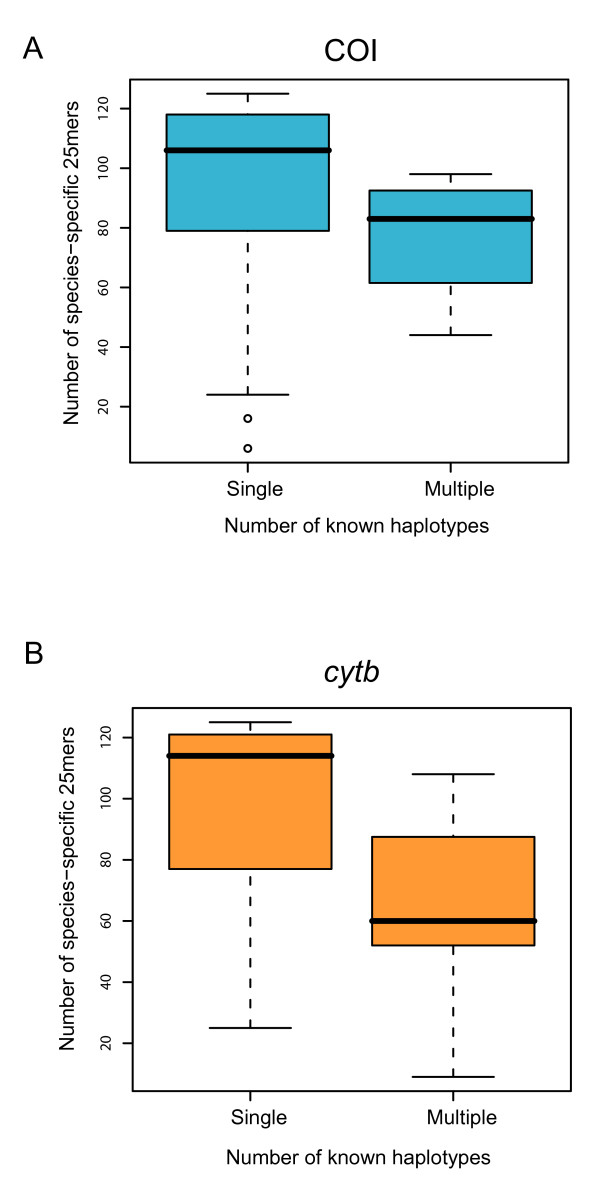
**Microarray probes**. Species with only one representative sequence were easy to design probes for. However, it is more challenging to find probes that were unique within species but capable of distinguishing between species when that species has several known haplotypes. Data is from 150 bases of the 5' region of COI (A) and *cytb *(B).

Both COI and *cytb *performed well as templates for probe design in mammalian dataset. However, we observed a sharp decline in the number of unique probes in species as haplotype diversity increased (Figure 1). For example, no 25mer probe in either COI or *cytb *was shared by all humans but also distinct from other species. We dealt with this limitation by choosing three probes from each species so that at least two probes should exactly match sequences within the target species. Another consideration was to ensure that the probes match among the first 150 bases from the 5' end of the target genes. This is important as COI and *cytb *are longer than 1 KB and are difficult to amplify in their entirety in samples with degraded DNA (i.e. traces of tissues, processed material and archival specimens) [10]. Our algorithm provided COI and *cytb *probes for 90.9% and 98.4% of the species in this mammalian dataset, respectively (Additional file 1). As for the bat dataset, we found a somewhat similar result (using available COI sequences) and were able to design probes for 89.7% of the species in this assemblage (Additional file 1).

### DNA barcoding analysis

**Figure 2:**
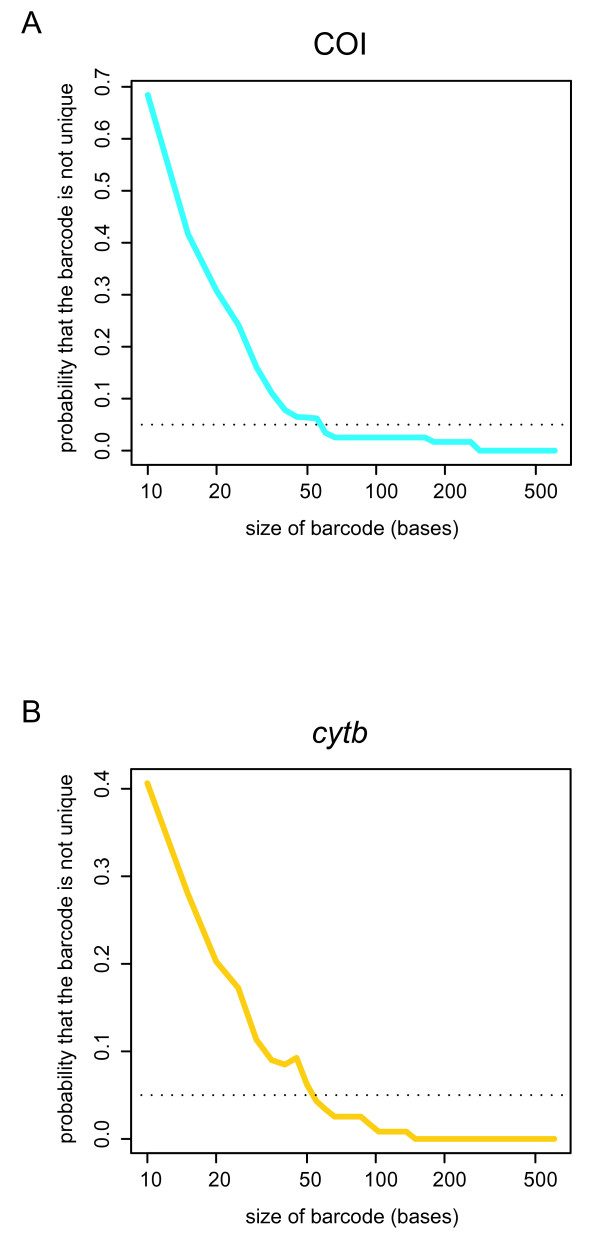
**Surprisingly short barcode sequences are capable of distinguishing between species**. Even 50-base barcodes can discriminate between >95% of species. The analysis is performed by adding sequence information from the 5' region of COI (A) and *cytb *(B) and calculating the probabilities.

Whole COI and *cytb *delivered similar results for the mammalian dataset, identifying all the species in our assemblage in a neighbor-joining (NJ) analysis [13] (Table 1). The standard animal barcode – a 650 bp fragment at the 5' end of COI – identified 96.7% of the species (Table 1). The same fragment size of *cytb *provided 98.3% species-level resolution (Table 1). Significantly, mini-barcodes of COI and *cytb *were also capable of discriminating among species of mammals, although the resolution was somewhat lower (Table 1). Interestingly, in the COI data we found that a mini-barcode positioned at nucleotides 437–654 (mini-barcode 5 in Table 1) provided the same resolution for species identification as the standard barcode sequence. In contrast, all the *cytb *mini-barcodes provided lower resolution as compared to a barcode-size fragment of the *cytb *gene (Table 1). The results obtained in the NJ analysis were confirmed when we plotted the sequence length against the probability of obtaining a unique sequence for each species. Interestingly, we found that the minimum signal required to provide unique barcodes in about 95% of the species in the mammalian dataset is a short ~50 base fragment of the 5' region of either the COI barcode or *cytb *gene, but the resolution decreases sharply with smaller sequences (Figure 2).

**Table 1:** COI DNA barcodes of varied lengths and comparable fragments of *cytb *are capable of identifying mammalian species in test assemblage of 1585 individuals from 121 species.

	**Length**	**% Res**	**% K2P**	**% Var**
**COI**				
Full gene	1557	100	14.9	56.3
Standard barcode	654	96.7	14.8	55.8
Mini-barcode 1	109	93.3	19.7	61.5
Mini-barcode 2	109	95	11.7	51.4
Mini-barcode 3	109	93.3	15.8	57.8
Mini-barcode 4	109	95	16.7	56.0
Mini-barcode 5	109	96.7	13.5	54.1
Mini-barcode 6	109	95	12.6	54.1
***cytb***				
Full gene	1149	100	16.9	69.3
Barcode size	654	98.3	15.9	65.9
Mini-barcode 1	109	95	15.7	67.9
Mini-barcode 2	109	93.3	17.3	65.1
Mini-barcode 3	109	96.7	16	68.8
Mini-barcode 4	109	95	13.8	58.7
Mini-barcode 5	109	95	15.3	65.1
Mini-barcode 6	109	95	18.2	69.7

Res, resolution in neighbor-joining analysis [13]; K2P, genetic distances based on Kimura two-parameter nucleotide substitution model [20]; var, variable sites.

**Figure 3:**
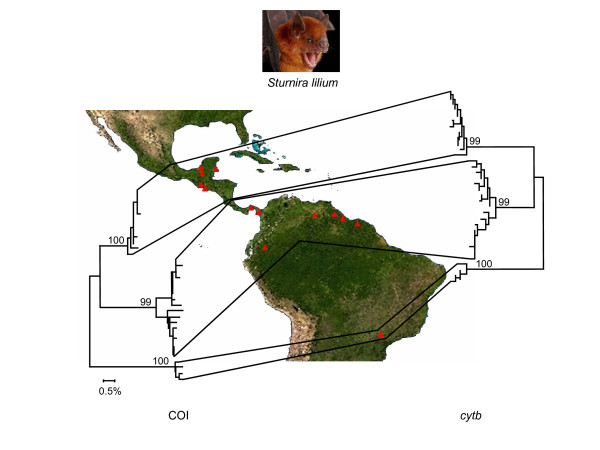
**DNA barcodes of COI and similarly sized sequences from *cytb *demonstrate three distinct geographically and genetically distinct groups within the neotropical bat species *Sturnira lilum***. Individual variation between cytochrome b sequences is greater than those within barcode sequences but resolution is internally consistent. The trees are assembled by using K2P genetic distances [20] in a neighbor-joining method [13]. Bootstrap values (1000 replicates) for major geographic lineages are shown above each branch. Photo of *Sturnira lilum *courtesy of Royal Ontario Museum.

An evaluation of COI barcodes in a dataset with lower taxonomic diversity (compared to our mammalian dataset) but with a somewhat higher density of sampling within a confined taxonomic assemblage – 840 individuals of 87 species of neotropical bats – showed a 100% resolution for species identification [14] (Table 2). Similar to the mammalian dataset, mini-barcodes of 109 bases were also capable of discriminating among more than 95% of the species in this bat dataset (Table 2). In addition, the minimum signal required to provide unique barcodes in more than 95% of the species of bats was a short ~30 base fragment of the 5' region of COI (results not shown). Comparison of COI and *cytb *in 34 individuals of one of these species, *Sturnira lilium*, across 13 sampling localities in Central and South America suggests that both genes provide similar resolution and can detect three geographical variants within this species (Figure 3). Similar resolution is achieved by using mini-barcodes of both COI and *cytb *for this species (results not shown).

**Table 2:** COI DNA barcodes of varied lengths are capable of identifying species in test assemblage of 840 individuals from 87 species of neotropical bats.

	**Length**	**% Res**	**% K2P**	**% Var**
**COI**				
Standard barcode	654	100	20.9	44.5
Mini-barcode 1	109	95.4	23.8	50.5
Mini-barcode 2	109	97.7	17.6	40.4
Mini-barcode 3	109	97.7	22.8	45.9
Mini-barcode 4	109	100	22.7	41.3
Mini-barcode 5	109	100	20.2	45.0
Mini-barcode 6	109	97.7	19.3	46.8

Res, resolution in neighbor-joining analysis [13]; K2P, genetic distances based on Kimura two-parameter nucleotide substitution model [20]; var, variable sites.

## Discussion

This study reveals that both arrays and barcodes are useful tools for the species-level identification of mammals. The main limitation of the array-based approach is that it requires advance knowledge of sequences in target species. Because of a lack of exact matches, undiscovered haplotypes or geographic variants could fail to anneal properly to the probes on the array. While we tried to avoid this problem by providing a set of three different probes per species, this factor can substantially limit the use of microarrays for large-scale biodiversity monitoring. Additionally, to explore unknown species in a given taxonomic group, it might be possible to design probe sets that specifically bind to members of a higher taxonomic level such as genus or family. However, in a situation such as *S. lilium*, with different geographical variants of up to 8% sequence variation in their COI/*cytb *genes, a probe set that is designed for the species in one locality might not bind to members of the species in other localities (results not shown). This hit or miss situation could make array technology less desirable in biodiversity monitoring across a wide geographic region. In fact, the current applications of microarrays are usually focused on a limited number of taxa [4]. Because of this, assembling a 'Mammalia Chip' might not be a feasible approach for biodiversity monitoring of all mammalian species.

Because barcoding is a sequencing-based technology, it avoids the problem of unknown haplotypes. New haplotypes can be compared to existing databases of barcodes, and they can be assigned to a particular species using probabilistic algorithms [15,16]. The final assignment of a new haplotype to a described species or its assignment to a new species will be achieved through comprehensive taxonomic analysis, which requires different types of data [17]. Our analysis supports this argument in all three datasets. While smaller fragments were less powerful in resolving some closely-related species, obtaining more sequence information in these cases (i.e. full-length barcode versus mini-barcode or the whole gene versus the barcode-size fragment) can increase the resolution [10]. However, while standard barcode-size fragments (650 bp) can be readily obtained in a single PCR amplification/sequencing from freshly collected or frozen tissue specimens, it is difficult to obtain 650-bp barcodes from specimens whose DNA is degraded (i.e. dried museum samples) [10]. The high effectiveness of mini-barcodes means that biomonitoring through barcodes can target different types of specimens, including museum samples or traces of tissues with degraded DNA [10]. The mini-barcode strategy also enables exploration of the use of massively parallel sequencing platforms, such as pyrosequencing-based [18] 454 Life Sciences sequencers, for barcoding applications. Interestingly, this technology uses an emulsion PCR approach for simultaneous amplification of several thousand 100–200 base DNA molecules in one reaction. This approach will therefore allow the use of mini-barcodes on environmental samples, which have traditionally been targets for array-based technology.

This study also provides evidence that both COI and *cytb *are useful species-level molecular markers for mammalian species. This finding is in agreement with earlier work [4]. However, when it comes to selecting a molecular marker, it is also important to consider operational issues such as the availability of robust PCR primers, standardization across a wide range of taxa, the robustness of amplifying shorter fragments in PCR reactions of degraded DNA, and the prevalence of mitochondrial nuclear pseudogenes. Our study further confirms that the standard COI barcode can be applied to mammalian species with a similar high species-level resolution as has been observed in other animal taxa tested.

## Conclusion

DNA-based methods such as DNA arrays and DNA barcodes provide substantial potential for biodiversity monitoring. However, as the scale of analysis increases, for example in large biodiversity surveys or analysis across wide taxonomic assemblages or different types of specimens, the scalability and sensitivity of these approaches become critical issues in their applicability. Our analyses using three different datasets of mammalian species spanning a wide range of taxa, suggest both DNA arrays and DNA barcodes provide high resolution (i.e. ~95%) across mammalian species. Because DNA arrays might fail to anneal to undiscovered haplotypes of a given species, their use is limited to taxa with known sequences. DNA barcoding, however, provides a higher flexibility for the identification of species in large taxonomic assemblages because it is based on obtaining sequence information that can be used for linking unknown haplotypes to known species.

## Methods

### Sequence information

We used COI and *cytb *genes for array-based and DNA barcoding analysis of mammalian species by using three taxonomic datasets. The first dataset was selected to allow comparison of the sequence information in the two genes from the same individuals of the same species in a wide taxonomic assemblage of mammals. We used all of the completely sequenced mitochondrial genome sequences of mammals to build this dataset. We downloaded the whole mitochondrial genome sequences of 1585 individuals from 121 mammalian species from GenBank (Additional file 2) and extracted the COI and *cytb *sequences from them. We refer to this dataset as the mammalian dataset. Our second dataset was selected to test the feasibility of arrays and barcodes in a dense and species-rich neotropical mammalian fauna: 840 individuals from 87 species of bats. This dataset included COI sequences from a recent barcode study on bats [14]. We refer to this dataset as the bat dataset. Finally, a third dataset was used as an extension to the bat dataset to compare the utility of both COI and *cytb *in DNA barcoding of 34 individuals of a single species of bat, *Sturnira lilium*, from a wide geographic range: 13 localities across nine countries in Central and South America. We refer to this dataset as the *S. lilium *dataset. Some COI and all *cytb *sequences for this third dataset were produced in this study (see Additional file 2 for GenBank accession numbers).

### Array-based analysis

For designing arrays, we chose COI and *cytb *as separate templates for a probe design algorithm. We assume that probes will be hybridized with amplicons from either COI or *cytb *of unknown specimens. Our algorithm searched for unique, species-specific sequences, but also considered intraspecific variation among haplotypes of each species (where different halpotypes were available). We designed probes that were 25 nucleotides long and hence suitable for Affymetrix-style single-channel microarrays (Additional file 1). Probes were chosen so that the theoretical probe-target melting temperatures fall within the range of 53.5–58°C, and the GC content falls within the range of 37–54.2%, as recommended by Pfunder et al [4]. We designed three probes for each species by using this algorithm (see below). We selected the first 150-bp sequences from the 5' end of each gene as a putative amplicon from which to select the probes (see below).

### DNA barcoding analysis

For DNA barcodes, we evaluated whole COI and *cytb *genes as well as various smaller fragments of the two as potential barcodes. For example, we analyzed the whole COI gene of 1557 bases and then performed the same analysis on a 654 base fragment of the 5' region of this gene – corresponding to the standard DNA barcode sequence – as well as smaller, equally-divided 109-bp fragments of the barcode region (i.e. positions 1–109, 110–218 and so on). A similar analysis was performed on *cytb *by selecting the 5' region of this gene as a potential 654 bp barcode-size region. We used this same analysis for both bat datasets. We counted the number of species with non-overlapping barcodes (i.e. barcodes that uniquely identify individuals of a species) in a neighbor-joining (NJ) analysis [13] as a measure of resolution [19]. In order to investigate the minimal sequence information required to perform DNA barcoding analysis, we plotted sequence length of putative COI barcodes and *cytb *gene (sequence information being added incrementally from the 5' end of gene) versus the probability of finding unique barcodes for each species.

## Authors' contributions

MH designed the project, performed DNA barcode analysis, and wrote the manuscript. GACS gathered sequence information from GenBank, designed and conducted DNA array analysis, and edited the manuscript. ELC carried out molecular methods, gathered sequence information of bats, helped with the analysis of barcode sequences, and edited the manuscript. PDNH aided the study design, provided tools/reagents, and edited the manuscript. All authors read and approved the final manuscript.

## Supplementary Material

Additional File 1 The list of species and the three probes for each of them. Click here for file

Additional File 2 **Table of completely sequenced mammalian mitochondrial genomes used in our analyses as well as COI and *cytb *genes sequenced in this study**. Shown are the species name, the common name, and the GenBank GI number for each sequence. Click here for file
