# Supplementary material for: Design and applicability of DNA arrays and DNA barcodes in biodiversity monitoring
Source: BMC Biol. 2007 Jun 13;5:24. doi: 10.1186/1741-7007-5-24 (PMC1906742; doi:10.1186/1741-7007-5-24)
Supplement: Additional File 1 — The list of species and the three probes for each of them. [file 1741-7007-5-24-S1.pdf]

Additional file 1.

The list of species and the three microarray probes for each of them. We have designed these probes such that at least one (and most commonly, two or three) of the probes in each set would light up. This variability in response is due to known haplotypes. For a handful of species in each table we were unable to design a unique probe set that was capable of distinguishing that species from closely-related neighbors. For example, Canus lupus (the wolf) is too closely related to Canus familiaris (dog) to reliably distinguish between them based on these very short sequences. The probes are contained within the first 150 bases of each gene, and are designed such that they will not cross-hybridize with any non-target part of any known mammalian mitochondrial genome. Probes have been chosen so that the theoretical probe-target melting temperatures fall within the range of 53.5C and 58C, and the GC content falls within the range of 37% and 54.2%, as recommended by Pfunder et al. (2004).

Pfunder, M., Holzgang, O. & Frey, J. E. 2004 Development of microarray-based diagnostics of voles and shrews for use in biodiversity monitoring studies, and evaluation of mitochondrial cytochrome oxidase I vs. cytochrome b as genetic markers. *Mol. Ecol.* **13**, 1277-86.

Additional file 1

Probe list for microarray analysis using 150 bp of 5' region of COI

**Species name**	**Probe 1**	**Probe 2**	**Probe 3**
Acinonyx jubatus	CTAGGTCAACCTGGCACACTACTAG	TAGGTCAACCTGGCACACTACTAGG	AGTAGGGACTGCTCTTAGTCTTCTA
Arctocephalus forsteri	ACCCTCTATTTACTATTCGGTGCAT	GTTAGGCCAACCAGGCACTCTACTA	TTGGCACCCTCTATTTACTATTCGG
Artibeus jamaicensis	GAGCAGGTATAGTAGGCACTGCACT	CCTATTATTTGGTGCTTGAGCAGGT	GTCTCCTTATTCGTGCAGAACTTGG
Balaena mysticetus	ACCAACCACAAAGATATTGGCACCT	CAACCACAAAGATATTGGCACCTTA	
Balaenoptera acutorostrata	GCACCCTGTATTTACTATTTGGTGC	ATAGTAGGCACCGGCCTAAGCTTAC	AAGCTTACTAATTCGCGCTGAGCTA
Balaenoptera bonaerensis	AATAGTAGGCACCGGCCTAAGCTTA	ATAGTAGGCACCGGCCTAAGCTTAC	AGCAGGAATAGTAGGCACTGGTCTA
Balaenoptera borealis	AACCACAAAGACATTGGTACCCTAT	ACTATTTGGTGCATGAGCAGGAATA	CAACCACAAAGACATTGGTACCCTA
Balaenoptera brydei	No sequences		
Balaenoptera musculus	No sequences		
Balaenoptera physalus	GCTAGGTCAACCTGGCACACTAATC	ACAAAGACATCGGCACCCTATACTT	AGCTAGGTCAACCTGGCACACTAAT
Berardius bairdii	GTCAACCAGGAACACTAATTGGGGA	AGGAATAGTGGGCACCGGTTTAAGT	AATCCGTACTGAATTAGGTCAACCA
Bos grunniens	No sequences		
Bos indicus	No sequences		
Bos taurus	No sequences		
Bubalus bubalis	CGGCACCCTATACTTACTATTTGGT	AAGATATCGGCACCCTGTACTTGCT	ATTAGGTCAACCTGGGACCCTACTC
Canis familiaris	ACTAGGTCAGCCCGGTACTTTACTA	ACTAGGTCAGCCCGGTACTTTACTA	TACTATTTGGAGCATGAGCCGGTAT
Canis latrans	No sequences		
Canis lupus	No sequences		
Caperea marginata	GAGCAGGAATAGTAGGTACTGGCCT	CACCCTATACTTATTATTCGGCGCC	CCCTATACTTATTATTCGGCGCCTG
Capra hircus	CAACCGCTGACTATTTTCAACCAAC	CATAGTAGGGACCGCCTTGAGCTTA	GAGCTTACTAATTCGCGCCGAACTA
Cavia porcellus	GAGCTGGTATAGTAGGTACTGCCCT	TAGTAGGTACTGCCCTAAGCTTGTT	CCCTAAGCTTGTTAATTCGAGCAGA
Cebus albifrons	ACAGGAACAGCCTTAAGTCTTCTAA	GCCTTAAGTCTTCTAATTCGAGCTG	AACAGGAACAGCCTTAAGTCTTCTA
Ceratotherium simum	GTACTCTCTACCTACTATTTGGCGC	TCGTTAACCGCTGACTGTTTTCAAC	CAACCAACCACAAGGACATTGGTAC
Chalinolobus tuberculatus	GCACCCTGTATCTTCTATTCGGTGC	ACAGCACTTAGCCTCTTAATTCGCG	GGTGGGTACAGCACTTAGCCTCTTA
Chlorocebus aethiops	TCTAAGTCTTCTCATTCGAGCTGAG	GGCACAGCTCTAAGTCTTCTCATTC	AGGCCAACCAGGKAGTTTACTAGGT
Chrysochloris asiatica	GACAACCTGGTGCACTATTAGGTGA	CTAATTCGGGCTGAACTAGGACAAC	AACTAGGACAACCTGGTGCACTATT
Colobus guereza	GGGCCGGAATAATGGGTATAGCTAT	GTCTTCTAATCCGAGCTGAACTAGG	CATGGGCCGGAATAATGGGTATAGC
Crocidura russula	CCTTAAGTATTTTAATCCGCGCTGA	ACTAACCACAAAGACATTGGCACAT	TCATAACTCGCTGACTCTTTTCCAC
Cynocephalus variegatus	AGTAGGAACAGCCTTAAGCCTTCTC	CAGCCTTAAGCCTTCTCATCCGAAC	GTAGGAACAGCCTTAAGCCTTCTCA
Dasypus novemcinctus	AGTCTACTAATTCGTGCCGAGCTTG	GAATAGTAGGCACCGCCCTAAGTCT	TATATTTACTATTTGGCGCCTGGGC
Dromiciops gliroides	GGCACCCTTTACTTACTATTTGGTG	TGAGCAGGAATAGTTGGTACAGCTC	CTCTAAGCCTATTAATCCGAGCTGA
Dugong dugon	ACAAAGACATTGGAACTCTGTACCT	ATCTTAATTCGAGCTGAGTTGGGCC	GTACCTACTATTTGGCGCTTGAGCC
Echinops telfairi	GGTACTGCTCTTAGCCTCCTAATCC	CACAGTAGGTACTGCTCTTAGCCTC	CAGTAGGTACTGCTCTTAGCCTCCT
Echinosorex gymnura	TAATTTTTGGTGCTTGAGCCGGTAT	TATAATTTTTGGTGCTTGAGCCGGT	ACGTCTCTCAGTCTGCTTATTCGAG

Elephantulus sp	GGAATAGTGGGGACTGCCCTAAGTA	CCACAAAGACATCGGGACCCTATAC	CAAAGACATCGGGACCCTATACTTA
Elephas maximus	TACTATTTGGTGCTTGAGCTGGTAT	AATTAGGTCAACCAGGCTCTCTTCT	TCTACTATTTGGTGCTTGAGCTGGT
Equus asinus	GTGCTGAATTAGGTCAACCTGGGAC	CCTAATCCGTGCTGAATTAGGTCAA	AATAGTAGGAACCGCCCTAAGCCTC
Equus caballus	CTCCTAATCCGTGCTGAATTAGGCC	ATAGTAGGAACTGCCCTAAGCCTCC	CATCAACCGCTGACTATTTTCAACT
Erinaceus europaeus	CTTGGCCAACCAGGAGCTTTATTAG	GCTTGGCCAACCAGGAGCTTTATTA	GAGCTTGAGCAGGTATAGTAGGCAC
Eschrichtius robustus	No sequences		
Eubalaena australis	TTATTATTTGGCGCCTGAGCAGGAA	ATTATTTGGCGCCTGAGCAGGAATA	ACCACAAAGACATTGGCACCTTATA
Eubalaena japonica	ATTTATTATTTGGCGCCTGAGCAGG	TTTATTATTTGGCGCCTGAGCAGGA	ATTTATTATTTGGCGCCTGAGCAGG
Eumetopias jubatus	AGCCTATTGATCCGCGCAGAATTAG	GAATTAGGCCAACCAGGCACTCTAC	CCTCTATCTACTATTCGGTGCATGA
Felis catus	CTCTTTACCTTTTATTCGGTGCCTG	TACCTTTTATTCGGTGCCTGAGCTG	GGTGGGGACTGCTCTTAGTCTTCTA
Galemys pyrenaicus	GTCTGTTAATTCGAGCTGAGTGGGG	TGCCCTAAGTCTGTTAATTCGAGCT	TTTATTTGGTGCCTGAGCTGGTATG
Gorilla gorilla	AACACTATATCTACTATTCGGCGCA	TCACCGACCGCTGATTATTCTCTAC	AACACTATATCTACTATTCGGCGCA
Halichoerus grypus	No sequences		
Hemiechinus auritus	TCACTCAGCTTACTAATTCGAGCTG	AGCAGGCATAGTAGGTACTTCACTC	GACAACCAGGAGCTCTAATGGGTGA
Herpestes javanicus	ACCACAAAGATATTGGCACCCTATA	AGTCTTTTAATTCGGGCCGAACTTG	CTGGTATAGTGGGAACTGCTCTTAG
Hippopotamus amphibius	TATATCTACTATTCGGCGCCTGAGC	ACTGGGTCAACCTGGCACACTATTA	ATATCTACTATTCGGCGCCTGAGCT
Homo sapiens	ACACTATACCTATTATTCGGCGCAT	ACCGTTGACTATTCTCTACAAACCA	CCGTTGACTATTCTCTACAAACCAC
Hylobates lar	No sequences		
Hyperoodon ampullatus	CTAACCACAAAGACATTGGCACTCT	CGCTGACTATTCTCAACTAACCACA	TAACCACAAAGACATTGGCACTCTA
Inia geoffrensis	AACTTGGACAACCCGGTACACTAAT	CATAGACCGTTGACTGTTCTCAACA	AGGAATAGTGGGTACTGGCTTAAGC
Isoodon macrourus	TAGGGACTGCTCTGAGCTTGTTAAT	CTATTTGGTGCCTGAGCAGGGATAG	GACTGCTCTGAGCTTGTTAATCCGA
Jaculus jaculus	CGGGATAATTGGTACTGCCTTGAGC	GTTCGTCACACGTTGACTTTTCTCA	GGATAATTGGTACTGCCTTGAGCAT
Kogia breviceps	CATAAAGACATCGGCACCTTGTATC	TAAAGACATCGGCACCTTGTATCTA	TAGTAGGCACTGGTTTGAGCCTACT
Lagenorhynchus albirostris	CACTGGCCTAAGCTTGTTGATTCGT	GCTGAATTAGGTCAACCTGGTACAC	ACTGGCCTAAGCTTGTTGATTCGTG
Lama pacos	TACTAATTCGAGCCGAATTAGGACA	ACAGGGCTAAGTCTACTAATTCGAG	AACCACAAAGATATCGGTACCCTCT
Lemur catta	CAGAACTAGGTCAACCTGGGTCTCT	CTCTCAGCCTTTTAATTCGAGCAGA	TGGTAGGAACAGCTCTCAGCCTTTT
Lepus europaeus	AATTAGGCCAACCTGGGACTTTACT	CACAAAGACATTGGAACTCTCTACC	TAGGAACAGCCCTAAGTCTGTTGAT
Loxodonta africana	ACCGCTGACTATATTCAACGAACCA	TATCCTAATTCGGGCAGAACTAGGC	TTCAACGAACCACAAAGATATCGGA
Macaca mulatta	CTATTTGGTGCATGAGCTGGAATCA	GCTCATTAATCGCTGACTCTTTTCA	CGCTGACTCTTTTCAACAAATCACA
Macaca sylvanus	ACTATTTGGCGCATGAGCTGGAATC	TTACTATTTGGCGCATGAGCTGGAA	CTATTTGGCGCATGAGCTGGAATCA
Macropus robustus	CCTTAAGTCTGCTCATTCGTGCAGA	ATTTGGTGCCTGAGCAGGTATAGTA	ATATCTCCTATTTGGTGCCTGAGCA
Macroscelides proboscideus	CATTCTTATCCGAGCCGAACTAGGT	TAGGTCAACCAGGCGCTCTATTAGG	GAGCATTCTTATCCGAGCCGAACTA
Mammuthus primigenius	ATCCTAATTCGGGCAGAACTAGGTC	CACAAAGACATTGGGACACTGTATC	TAATTCGGGCAGAACTAGGTCAACC
Megaptera novaeangliae	AAGCTTATTAATTCGCGCTGAGCTA	AGCTTATTAATTCGCGCTGAGCTAG	TTATTAATTCGCGCTGAGCTAGGTC
Mogera wogura	ATAGCTGGTACCGCTCTAAGTCTGC	GTGCTGAATTAGGACAACCAGGTAC	GCTAATTCGTGCTGAATTAGGACAA
Monodon monoceros	ATCACAAGGACATTGGCACCCTATA	CGGCCTAAGCTTATTAATTCGTGCT	GAACCGGCCTAAGCTTATTAATTCG
Muntiacus crinifrons	AGCAGGCATAGTAGGAACAGCTCTA	GCATAGTAGGAACAGCTCTAAGCCT	TCATAAAGACATCGGCACCCTCTAC

Muntiacus muntjak	CATAGTAGGGACAGCCCTAAGCCTA	CCCTAAGCCTATTAATTCGTGCTGA	CCATAAAGACATCGGCACCCTGTAC
Muntiacus reevesi	CAGGCATAGTAGGAACAGCCCTAAG	CCGCTGATTATTTTCAACCAATCAC	AGCCTGTTAATTCGTGCTGAACTGG
Mus musculus molossinus	TAGTAGGCACCGCACTAAGTATTTT	ATTTGGAGCCTGAGCGGGAATAGTA	ACTATTTGGAGCCTGAGCGGGAATA
Mus musculus	ACCCTCTATCTACTATTCGGAGCCT	CTATCTACTATTCGGAGCCTGAGCG	GGGAATAGTGGGTACTGCACTAAGT
Myoxus glis	CTACTATTTGGTGCTTGAGCCGGAA	ACAGCCTTAAGTCTCTTAATCCGTG	AAATCACAAAGACATTGGCACGCTA
Nannospalax ehrenbergi	ACCAACCATAAGGATATCGGAACAC	CTTAATCCGAGCAGAACTTGGACAA	ATCTTAATCCGAGCAGAACTTGGAC
Nasalis larvatus	GGAACCGCAGGTATAGCTATAAGCC	CGCTGGCTATTCTCCACAAATCATA	CCGCTGGCTATTCTCCACAAATCAT
Nycticebus coucang	TTTGCTAATCCGAGCAGAGCTAGGT	GTTCATTAACCGCTGGCTCTATTCT	CGCTGGCTCTATTCTACCAATCACA
Ochotona princeps	CGGCACCCTATACATACTATTCGGC	TATACATACTATTCGGCGCTTGGGC	ATCAACCGTTGATTGTTCTCCACAA
Odobenus rosmarus rosmarus	ACAAATCACAAGGACATCGGCACTC	GCACTCTCTATTTATTATTCGGCGC	ACTAGGTCAACCTGGCACTCTATTA
Ornithorhynchus anatinus	TGAATTAGGTCAACCCGGTTCATTA	ATCTTCTATTTGGTGCATGAGCTGG	CCGGCACAGCCCTTAGTATCCTAAT
Orycteropus afer	CCCTAAGCTTATTAATCCGAGCTGA	ACAGCCCTAAGCTTATTAATCCGAG	ACTAGGTCAGCCTGGTGCTCTACTA
Oryctolagus cuniculus	AAGACATCGGCACTCTTTATCTCCT	TGTTCGTCAATCGTTGACTTTTCTC	TCCTATTTGGAGCTTGAGCTGGGAT
Ovis aries	CAACCAACCACAAAGATATCGGCAC	TATCGGCACCCTTTACCTTCTATTT	GGAACCGCCTTAAGCCTACTAATTC
Pan paniscus	AGCCCTAAGTCTCCTTATTCGAGCT	AGCCCTAAGTCTCCTTATTCGAGCT	AGCCCTAAGTCTCCTTATTCGAGCT
Papio hamadryas	AGGCATGGCCCTAAGTCTTCTCATT	AGTTACAGGCATGGCCCTAAGTCTT	ATACCTGTTATTTGGTGCATGAGCC
Phoca vitulina	No sequences		
Physeter catodon	AACCATAAGGACATCGGCACTCTAT	AGGACATCGGCACTCTATATCTACT	TATCTACTATTCGGTGCCTGAGCGG
Pipistrellus abramus	ATTAGGTCAACCAGGAGCTTTGCTT	CCTTTACTTACTATTTGGCGCCTGG	TGCACTAAGTCTATTAATTCGTGCC
Platanista minor	TTTGGTGCTTGAGCAGGGATAGTAG	GTAGGTACCGGCCTAAGCTTACTAA	GGATAGTAGGTACCGGCCTAAGCTT
Pongo pygmaeus abelii	ATCGGGACACTATACCTGTTATTCG	GCTATTCTCCACGAACCACAAAGAC	ACCACAAAGACATCGGGACACTATA
Pongo pygmaeus	CTCCACGAACCACAAAGATATTGGA	CTAAGCCTCCTCATTCGTGCTGAAC	TCTCCACGAACCACAAAGATATTGG
Pontoporia blainvillei	ACCGCTGATTATTCTCAACAAACCA	GGCACCGGCCTAAGTCTATTAATCC	ACTATTCGGTGCTTGAGCAGGAATA
Presbytis melalophos	TGGCTATAAGCCTCCTTATTCGAGC	AAGCCTCCTTATTCGAGCTGAGCTA	TATAAGCCTCCTTATTCGAGCTGAG
Procavia capensis	CCGTTGATTGTTTTCAACCAACCAT	TGGAGCTTGAGCTGGAATAGTAGGA	CATCAACCGTTGATTGTTTTCAACC
Procolobus badius	ATTTATTATTCGGAGCATGGGCTGG	CCTTCTAATCCGAGCCGAACTAGGT	ATAAGCCTTCTAATCCGAGCCGAAC
Pteropus scapulatus	GTTGACTCTTTTCAACCAACCACAA	CGTTGACTCTTTTCAACCAACCACA	TGACTCTTTTCAACCAACCACAAAG
Pygathrix nemaeus	CCTTATTCGAGCTGAACTAGGCCAG	AGCTGGAACCACAGGTATAGCTATA	ATGAGCTGGAACCACAGGTATAGCT
Pygathrix roxellana	CTTATTCGAGCTGAACTAGGTCAAC	AACCATGGGTATGGCTATAAGTCTC	CTAGGTCAACCTGGCAACCTGTTAG
Rattus norvegicus	ACCGTTGACTCTTTTCAACTAACCA	TTTGGAGCCTGAGCAGGAATAGTAG	ACTAACCACAAAGATATCGGAACCC
Rhinoceros unicornis	AATAGTAGGAACCGCCCTAAGCCTT	CACTCTATACCTGTTATTTGGCGCC	TCTAATTCGCGCCGAATTAGGTCAG
Rhinolophus monoceros	GGCACCCTCTATTTACTATTTGGTG	ACCGCTCTAAGCCTACTCATTCGAG	CACCCTCTATTTACTATTTGGTGCC
Rhinolophus pumilus	GCACCCTCTACTTACTATTTGGTGC	AAGCCTGCTCATTCGAGCTGAATTA	CAAAGACATCGGCACCCTCTACTTA
Sciurus vulgaris	TACTAATTCGAGCTGAACTGGGTCA	ATATCTCTTATTTGGTGCCTGAGCT	TCTCTTATTTGGTGCCTGAGCTGGA
Semnopithecus entellus	AGCTGGAACTGCAGGCATAGCTATA	ATAAGTCTTCTCATCCGAGCCGAAT	AACTGCAGGCATAGCTATAAGTCTT
Sus scrofa	TCAACAAACCACAAAGACATCGGCA	CCTACTAATTCGCGCTGAACTAGGT	TACTAATTCGCGCTGAACTAGGTCA
Tachyglossus aculeatus	TCTCATTCGATCCGAATTAGGCCAA	TCATTCGATCCGAATTAGGCCAACC	ACAGCCCTCAGTATTCTCATTCGAT

Talpa europaea	TGAACTAGGACAACCAGGTACTCTA	TCAACAAACCACAAAGATATCGGCA	GTGCTGAACTAGGACAACCAGGTAC
Tamandua tetradactyla	ACAAATCATAAAGATATCGGCACCC	GTACAGGCCTAAGCATCCTTATCCG	TTGGTACAGGCCTAAGCATCCTTAT
Tarsius bancanus	TGAGCTGGAATAGTAGGAACAGCCC	GAGCTGGAATAGTAGGAACAGCCCT	CTGAGCTGGAATAGTAGGAACAGCC
Thryonomys swinderianus	AGGAATAGTAGGAACTGCTCTAAGC	GAACTGCTCTAAGCCTTTTAATCCG	TTAGGACAACCGGGTACCCTACTAG
Thylamys elegans	AAGTCTTCTAATCCGAGCCGAACTT	TACCTAATTTTTGGGGCTTGAGCAG	ATTTTTGGGGCTTGAGCAGGTATAG
Trachypithecus obscurus	ATGAGCTGGAACCGTAGGTATAGCT	ATAAGTCTCCTTATTCGAGCCGAAC	AACCGCTGGTTATTCTCTACAAACC
Trichosurus vulpecula	CCGCTCTAAGTCTATTAATTCGCGC	ATAACAGGCACCGCTCTAAGTCTAT	TGGTCAACCAGGAACTCTTATTGGC
Tupaia belangeri	AGCCTTAAGTCTTCTTATTCGCGCC	ATAGTCGGAACAGCCTTAAGTCTTC	CCGGAATAGTCGGAACAGCCTTAAG
Urotrichus talpoides	GAGCCTACTAATTCGTGCCGAACTC	CTGAGCCTACTAATTCGTGCCGAAC	CTACTAATTCGTGCCGAACTCGGTC
Ursus americanus	ATGAGCCGGAATAGTAGGTACTGCT	GGTGCATGAGCCGGAATAGTAGGTA	GAGCCGGAATAGTAGGTACTGCTCT
Ursus arctos	ACATTGGCACTCTTTACCTTCTGTT	AAGCCGGTGATTATTCTCTACGAAC	AAAGACATTGGCACTCTTTACCTTC
Ursus maritimus	CTTTATCTTCTGTTCGGTGCATGAG	TTATCTTCTGTTCGGTGCATGAGCC	CCCTTTATCTTCTGTTCGGTGCATG
Vombatus ursinus	CTGTACCTCTTATTCGGTGCCTGAG	GCAGGAATAGTAGGGACAGCCCTAA	ACAGCCCTAAGCCTATTAATTCGAG

**Probe list for microarray analysis using 150 bp of 5' region of cytb**			
**Species name**	**Probe 1**	**Probe 2**	**Probe 3**
Acinonyx jubatus	GTCTGCCTAGTCCTACAGATCCTAA	CTACTAGGAGTCTGCCTAGTCCTAC	TAGTCCTACAGATCCTAACCGGCCT
Arctocephalus forsteri	TCCGAAAAATACATCCACTGGCCAA	TAGCCTTACAAATCCTAACAGGCCT	CATCCACTGGCCAAAATTATCAACA
Artibeus jamaicensis	GAGTTTGTTTGGGCGTACAAATTCT	GCTCCCTCAAGCCTTTCATCATGAT	CAATAGCTCATTCGTAGACCTTCCT
Balaena mysticetus	GGCCTTTGCCTGATTATACAAATCC	TCCCTACTTGGCCTTTGCCTGATTA	TGGCCTTTGCCTGATTATACAAATC
Balaenoptera acutorostrata	ATGAAACTTTGGCTCTCTACTCGGC	CGATCTACCCACCCCATCAAACATC	ATGAAACTTTGGCTCTCTACTCGGC
Balaenoptera bonaerensis	GATGAAACTTTGGCTCTCTACTCGG	TCTACCCACCCCATCAAACATCTCC	ACCCACACCATCAAACATCTCCTCA
Balaenoptera borealis	GATTGTCAACGATGCATTCGTTGAT	TCAACGATGCATTCGTTGATCTCCC	AGATTGTCAACGATGCATTCGTTGA
Balaenoptera brydei	AACGATGTATTCGTTGATCTCCCCA	GTCAACGATGTATTCGTTGATCTCC	TGTCAACGATGTATTCGTTGATCTC
Balaenoptera musculus	CATTGATCTCCCTACCCCATCAAAC	GCATTCATTGATCTCCCTACCCCAT	CCCTACCCCATCAAACATCTCCTCA
Balaenoptera physalus	CCTACTCGGCCTCTGCTTAATTATA	TTCATGATGGAACTTCGGCTCCCTA	AAAAATCGTCAACGACGCATTCGTC
Berardius bairdii	GCCTTTGCCTAATCATGCAAATTCT	CGGCCTTTGCCTAATCATGCAAATT	CCTAATCATGCAAATTCTCACAGGC
Bos grunniens	CATTGACCTTCCAGCTCCATCAAAC	CCCTCCTAGGAGTATGCTTAATCCT	CATTCATTGACCTTCCAGCTCCATC
Bos indicus	TCATGATGGAATTTCGGTTCCCTCC	CATGATGGAATTTCGGTTCCCTCCT	CATGATGGAATTTCGGTTCCCTCCT
Bos taurus	TCGAAAGTCCCACCCACTAATAAAA	TCGAAAGTCCCACCCACTAATAAAA	GCCTAATCCTACAAATCCTCACAGG
Bubalus bubalis	CCTGCTCCATCAAACATCTCATCAT	CTCTCTCCTAGGCATCTGCCTAATC	GCATCTGCCTAATCCTGCAAATCCT
Canis familiaris	CCACCCACTAGCCAAAATTGTTAAT	AACATCTCTGCTTGATGGAACTTCG	TCGGATCCTTACTAGGAGTATGCTT
Canis latrans	AACATTCGAAAAACTCACCCACTAG	TTCGGATCCTTGCTAGGAGTATGCC	CACCCACTAGCCAAAATTGTCAATA
Canis lupus	No sequences		
Caperea marginata	GCATTCATTGATCTTCCCACCCCAT	TTCCCACCCCATCAAATATCTCCTC	TGGCCTTTGCCTAATCATACAAATC
Capra hircus	ATTTATTGACCTCCCAACCCCATCA	TCCGAAAGACCCACCCATTAATAAA	CCAACCCCATCAAACATCTCATCAT
Cavia porcellus	TAACCACTCCCTAATTGACCTCCCA	GATGAAACTTCGGCTCCCTCTTAGG	CTAGGCCTGCAAATTATTACAGGAC

Cebus albifrons	TTATTGATCTGCCCACACCATCCAA	CCCGCAAAACACACCCATTAATAAA	CATCTCCTCCTGATGAAACTTCGGA
Ceratotherium simum	AACATCCGTAAATCCCACCCACTAA	CCGTAAATCCCACCCACTAATCAAA	CAAACATCTCAGCCTGGTGAAATTT
Chalinolobus tuberculatus	TGTTTAGCGTTACAAATCCTGACAG	TTCATTGATTTACCCACCCCATCTA	AATTTTGGATCCCTTCTAGGCACCT
Chlorocebus aethiops	TAATCAACCACTCCCTCATTGACCT	TCCATATGATGAAACTTCGGCTCAC	ACCCCAATACGTAAATCCAACCCAA
Chrysochloris asiatica	GCTCACTACTAGGCCTTTGCTTAAT	CCAGCACCATCTAACATTTCCGCAT	CATTTCCGCATGATGAAACTTCGGC
Colobus guereza	TTAACCATGCTTTCATCGACTTACC	ACCTCAACACGTAAGTCTAACCCAA	CCTCAACACGTAAGTCTAACCCAAT
Crocidura russula	CAGCACCCTCAAACATTTCATCATG	ACTCGGAATCTGCTTAATCGCACAA	CTCGGAATCTGCTTAATCGCACAAA
Cynocephalus variegatus	AAATCCCACCCTCTCCTAAAAATCA	TCATTCATTGACCTTCCCACACCAT	AAATTTCGGCTCACTACTAGGTACA
Dasypus novemcinctus	AACCACCCACTCCTAAAAATCGTAA	AACATCCGTAAAAACCACCCACTCC	ACCAACATCCGTAAAAACCACCCAC
Dromiciops gliroides	ACCTGCCTTGTAATCCAAATCCTAA	GACTTACCAGCACCCTCCAATATCT	TGACTTACCAGCACCCTCCAATATC
Dugong dugon	GGGCATGCCTGATTATTCAAATTCT	CCTAAACAACTCCTTCATTGACCTC	CCTCCCTACCCCCGTAAATATCTCA
Echinops telfairi	ACTAGCCAAGATCATCAACAGTTCC	AGCCAAGATCATCAACAGTTCCTTC	CTAGCCAAGATCATCAACAGTTCCT
Echinosorex gymnura	TTATTGACCTACCAACCCCATCAAA	AAATTTCGGCTCACTACTAGGCCTA	ACTAACATACGAAAAACCCACCCGT
Elephantulus sp	CTCCTCATGATGAAACTTCGGCTCA	ACTCCTGGGCTTATGCCTAATGATC	ACTCCTGGGCTTATGCCTAATGATC
Elephas maximus	CTCACTACTAGGAGCGTGCCTAATT	ACTAGGAGCGTGCCTAATTACCCAA	CACCCCCTGTTTAAAATCATCAACA
Equus asinus	TCCTCCAAATCCTAACAGGCCTATT	GAAACTTTGGCTCCCTCCTAGGAAT	ATCACTCTTTTATCGACCTGCCAAC
Equus caballus	CTAATCCTCCAAATCTTAACAGGCC	TCCGGAAATCTCACCCACTAATTAA	AAACATCCGGAAATCTCACCCACTA
Erinaceus europaeus	CTAGGCCTATGCCTAATTACCCAGA	TGGTTCATTACTAGGCCTATGCCTA	TACTAGGCCTATGCCTAATTACCCA
Eschrichtius robustus	TCCCTACTTGGCCTCTGCTTAATTA	CCCTACTTGGCCTCTGCTTAATTAT	AAATCATCAACGACGCATTCGTCGA
Eubalaena australis	No sequences		
Eubalaena japonica	TATTAACGACGCCTTCATCGATCTC	TCCCCACTCCATCAAATATCTCTTC	TCTCCCCACTCCATCAAATATCTCT
Eumetopias jubatus	TGAAACTTTGGATCCCTCCTTGCAG	CAGCATGCTTAGCCTTACAAATCTT	ATGACCAACATTCGAAAAGCACATC
Felis catus	CTAGGAGTCTGCCTAACCTTACAAA	AGTCTGCCTAACCTTACAAATCCTC	TGCCTAACCTTACAAATCCTCACCG
Galemys pyrenaicus	TCGGTTCCCTACTAGGAATTTGCCT	CCCTACTAGGAATTTGCCTAGTGCT	TTCGGTTCCCTACTAGGAATTTGCC
Gorilla gorilla	GTGCCTGCTTAATCCTTCAAATCAC	CTCACTCCTTGGTGCCTGCTTAATC	TCCTTCAAATCACCACAGGGCTATT
Halichoerus grypus	TCGACCTACCCACACCGTCAAATAT	AACAACTCATTTATCGACCTACCCA	TTCTCGGAATCTGCCTAATCCTACA
Hemiechinus auritus	TCTTTCATTGACCTCCCTACCCCAT	GACCTCCCTACCCCATCAAATATTT	ATTCTTTCATTGACCTCCCTACCCC
Herpestes javanicus	CACACCCACTCATTAAAATCGTCAA	AAACTTTGGCTCCCTTTTAGGAGTG	ACTTTGGCTCCCTTTTAGGAGTGTG
Hippopotamus amphibius	CTTGGCGTCTGCCTAATCCTACAAA	CAGCTCCATCAAACATCTCATCGTG	AACATCCGAAAATCTCACCCCTTAA
Homo sapiens	CAACATCTCCGCATGATGAAACTTC	CCTGATCCTCCAAATCACCACAGGA	TGATCCTCCAAATCACCACAGGACT
Hylobates lar	TCCAGCCCCATCCAACATTTCTATA	CCCTGCGCAAAACTAACCCACTAAT	ATCAACCACTCACTTATCGACCTTC
Hyperoodon ampullatus	ATCACACAAATTCTCACAGGCCTAT	TCGGCCTCTGTCTAATCACACAAAT	CTCGGCCTCTGTCTAATCACACAAA
Inia geoffrensis	CCTACTCGGTCTCTGCCTAATTATT	ATAATGCACTCATTGACCTCCCCAC	TTCCCTACTCGGTCTCTGCCTAATT
Isoodon macrourus	ATCAAACATCTCAGCATGATGGAAC	TTATCGATTTACCTGCCCCATCAAA	GCCCCATCAAACATCTCAGCATGAT
Jaculus jaculus	CAACATCCGTAAGACTCACCCACTC	TTCAGCATGATGAAACTTTGGCTCT	ATGACCAACATCCGTAAGACTCACC
Kogia breviceps	CTTGGCCTGTGTCTCATCACACAAA	AATCGTCAACAACGCATTCATCGAC	TGGCCTGTGTCTCATCACACAAATC
Lagenorhynchus albirostris	GACGCATTCATTGACCTACCCACTC	GAAATTTTGGCTCCCTACTAGGCCT	TGACCTACCCACTCCATCTAGTATC

Lama pacos	ATGACCAATATCCGAAAGTCCCACC	ATCTTCCAGCCCCTTCTAACATCTC	TCCAGCCCCTTCTAACATCTCATCA
Lemur catta	CCCTACAAATCATCACAGGGCTATT	CATTCATTGACCTCCCAACACCATC	AATTTCGGTTCCCTTCTAGGAGCCT
Lepus europaeus	AAACTTCGGCTCTCTATTGGGATTA	AACCACTCTCTAATCGACCTTCCCG	GTAAAACGCACCCCCTACTAAAAAT
Loxodonta africana	ATCCTTCATTGATCTACCTACCCCA	GATCTACCTACCCCATCCAACATCT	ATTGATCTACCTACCCCATCCAACA
Macaca mulatta	AATACGCAAATCCAACCCAATCCTA	CCCAACCTCTCCATATGGTGAAACT	ATACGCAAATCCAACCCAATCCTAA
Macaca sylvanus	ACTTCGGTTCACTTCTTACAGCTTG	TGCCTAATCCTGCAAATCATCACAG	CTAATCCTGCAAATCATCACAGGCC
Macropus robustus	ACCTACGTAAATCCCACCCCCTAAT	ATTTCAGCCTGATGAAACTTCGGCT	GGAGCCTGCCTAATTATCCAAATCC
Macroscelides proboscideus	ACACCCATTACTCAAAATCCTCAAC	TGCTCCATCTAACATTTCATCCTGA	CACACCCATTACTCAAAATCCTCAA
Mammuthus primigenius	TTCGAAAATCTCACCCCCTACTTAA	ACATTCGAAAATCTCACCCCCTACT	TCGAAAATCTCACCCCCTACTTAAA
Megaptera novaeangliae	CATTGATCTACCCACCCCATCAAAT	ACGACACATTCATTGATCTACCCAC	TGATCTACCCACCCCATCAAATATC
Mogera wogura	TCGAAAGACCCACCCACTAATAAAA	TCGGATCATTACTTGGAATCTGCCT	ACATTCGAAAGACCCACCCACTAAT
Monodon monoceros	TTCATGATGAAACTTTGGCTCCCTA	TAACAGCACATTCATCGATCTCCCT	ACAGCACATTCATCGATCTCCCTAC
Muntiacus crinifrons	GAAACTTCGGCTCCCTACTAGGAAT	GCCCCATCAAACATCTCATCCTGAT	AACTTCGGCTCCCTACTAGGAATTT
Muntiacus muntjak	GTAAACAACGCATTCATTGACCTCC	CAGCCCCATCAAACATTTCATCTTG	TGTAAACAACGCATTCATTGACCTC
Muntiacus reevesi	TGACAAATATCCGAAAAACCCACCC	ACAAATATCCGAAAAACCCACCCAC	CAAATATCCGAAAAACCCACCCACT
Mus musculus molossinus	AACCACTCATTCATTGACCTACCCG	CTTTGGGTCCCTTCTAGGAATCTGC	GGGTCCCTTCTAGGAATCTGCCTAA
Mus musculus	TTCTAGGAGTCTGCCTAATAGTCCA	TCTAGGAGTCTGCCTAATAGTCCAA	TCCCTTCTAGGAGTCTGCCTAATAG
Myoxus glis	TACCAACCCCTTCAAATATTTCAGC	AGCCTGCTTAGGAATTCAAATCCTA	TCGAAAATCCCACCCTCTCATCAAA
Nannospalax ehrenbergi	GCAAATCACACCCCCTAATCAAAAT	CTTCGGATCCCTACTAGGAGTATGT	CACTCATCGACTTACCAACACCATC
Nasalis larvatus	ACATTTCCATGTGATGAAACTTCGG	TTGATTTACCCACCCCATCAAACAT	GATGAAACTTCGGCTCTCTTCTAGC
Nycticebus coucang	CCCTGCACCATCTAACATCTCATCT	TCTCCCTGCACCATCTAACATCTCA	GGTTCATTACTGGGACTATGTCTGA
Ochotona princeps	TGATGAAACTTCGGATCCCTCCTAG	CCACTCTTTTATCGACCTGCCAACC	GCATCCAAATCATCACCGGCCTATT
Odobenus rosmarus rosmarus	TAATTCTACAAATCCTAACGGGCCT	TTTATCGACTTACCCACACCATCAA	ACATTTATCGACTTACCCACACCAT
Ornithorhynchus anatinus	TTTTGGCTCTCTTTTAGGCCTCTGC	GCCTAATTATCCAAATCCTCACAGG	GGCCTCTGCCTAATTATCCAAATCC
Orycteropus afer	CTTGGAATCTGCCTCATCATCCAAA	TGACCTTCCAACTCCTTCTAACATC	AATCTGCCTCATCATCCAAATTGTC
Oryctolagus cuniculus	TTCCTGCTCCATCAAACATCTCTGC	CACTCCCTAATTGACCTTCCTGCTC	AATTGACCTTCCTGCTCCATCAAAC
Ovis aries	TGGCTCTCTCCTAGGCATTTGCTTA	TGATCTCCCAGCTCCATCAAATATT	TCTCCCAGCTCCATCAAATATTTCA
Pan paniscus	ACCCCATCCAATATTTCCACATGAT	CCCACCCCATCCAATATTTCCACAT	ACCCCATCCAATATTTCCACATGAT
Papio hamadryas	CATCCAACATCTCCATCTGATGGAA	CACTCCTTTATCGACCTACCTACCC	AATTTCGGCTCACTTCTTGCAACCT
Phoca vitulina	CCCACACCATCAAATATCTCGGCAT	TCTCGGCATGATGAAACTTTGGATC	TACCCACACCATCAAATATCTCGGC
Physeter catodon	TGGACTCTGCCTGATCATACAAATC	TGATCATACAAATCCTAACAGGCCT	ATTTCCTCATGATGAAACTTCGGCT
Pipistrellus abramus	CATCCGAAAATCCCACCCATTAATT	TCCCTTTTAGGCATTTGTTTAGCAC	AACAGCTCATTCATTGACCTACCAA
Platanista minor	ATCTCCTCTTGATGAAACTTTGGCT	CATTGATCTCCCAACCCCATCAAAC	TGATCTCCCAACCCCATCAAACATC
Pongo pygmaeus abelii	TCAACCACTCACTTATCGACCTCCC	TGCTTAATCATCCAAATCACCACTG	ATCATCCAAATCACCACTGGACTAT
Pongo pygmaeus	GCCTGCTTAATCATCCAAACCATCA	TAATCATCCAAACCATCACTGGACT	ATCAAACATCTCTGCATGATGGAAC
Pontoporia blainvillei	CTCTTCATGATGAAACTTCGGCTCA	CAATAACGCATTCATCGACCTTCCT	CCTACCCCATCAAACATCTCTTCAT
Presbytis melalophos	TCCCTACTAGCAACCTGCTTAATTT	ACTCCCTTATTGATTTACCTACCCC	CTCCCTACTAGCAACCTGCTTAATT

Procavia capensis	CCACTACTTAAAACCATCAACGACG	ATTGATCTACCAACGCCATCCAACA	CTTAAAACCATCAACGACGCCTTCA
Procolobus badius	AACCTGCCTGCTCCTACAAATTATT	CACGCTCTTATTGATTTACCTACCC	CTACCCCTCCCAATATCTCTACATG
Pteropus scapulatus	ATCAACGACTCGCTAATCGACTTAC	AAACTTCGGCTCACTATTAGGCATC	CTGCCTAGCCATCCAAATTTTAACA
Pygathrix nemaeus	TTGATCTACCAACCCCATCAAACAT	TCCCCATACGCAAATCTAATCCAAT	CTACCAACCCCATCAAACATCTCAG
Pygathrix roxellana	TCTGCATGATGAAACTTCGGTTCCC	CATGATGAAACTTCGGTTCCCTTTT	TCGGTTCCCTTTTAGCAACTTGTTT
Rattus norvegicus	GCCTCATAGTACAAATCCTCACAGG	CTTCGGTTCTCTACTAGGAGTATGC	CATCCGAAAATCTCACCCCCTATTC
Rhinoceros unicornis	TTCATCGACCTACCTACCCCATCAA	CTACCCCATCAAACATCTCATCTTG	CTCATTCATCGACCTACCTACCCCA
Rhinolophus monoceros	AACATTCGTAAATCCCACCCACTAT	AGATCATCAACGACTCGTTCATCGA	CAACATTCGTAAATCCCACCCACTA
Rhinolophus pumilus	CCAACATTCGTAAATCCCACCCATT	TTCATCGACTTACCAGCCCCATCAA	GACTCATTCATCGACTTACCAGCCC
Sciurus vulgaris	TTTTATTGACCTCCCAGCTCCCTCA	TATCCGCAAAACCCACCCTCTAATC	CTCAAACATCTCAGCCTGATGAAAC
Semnopithecus entellus	TGAAACTTCGGTTCTCTACTAGCAA	ACCATTCCCTTACTGACTTACCCAC	GTTCTCTACTAGCAACCTGCTTGAT
Sus scrofa	GCCTAATCTTGCAAATCCTAACAGG	CCCTCTTAGGCATCTGCCTAATCTT	TAATCTTGCAAATCCTAACAGGCCT
Tachyglossus aculeatus	GGCATATGCCTTATCGTCCAAATCC	AACTTTGGCTCCCTACTAGGCATAT	CTTATCGTCCAAATCCTCACAGGCC
Talpa europaea	CAACAGCTCATTCATTGACCTACCA	ATGACAAACATCCGAAAAACACACC	AGCTCATTCATTGACCTACCAGCAC
Tamandua tetradactyla	CATCAACCAATCATTCATCGACCTC	CGACCTCCCTACACCATCAAACATT	TCTGCCTAGTAATCCAAACCCTAAC
Tarsius bancanus	CTGCTTAGGACTTCAAATCGTCACA	CAAATTTACGAAAAACTCACCCCCT	TGAAACTTTGGCTCTCTCTTAGGGG
Thryonomys swinderianus	TCATTGATTTACCCACACCACCAAA	GCGCTTGCTTAGCTTTACAAATCCT	CCGAAAAGCCCACCCACTACTAAAA
Thylamys elegans	ATTTTGGGTCTCTCCTAGGAATCTG	CAGCTTGATGGAATTTTGGGTCTCT	CATTTCAGCTTGATGGAATTTTGGG
Trachypithecus obscurus	TGACCCCCCTACGTAAATCTAATCC	ACTTACCCACCCCATCAAACATTTC	CCCATCAAACATTTCCACATGATGA
Trichosurus vulpecula	CCAACATCTCTGCCTGATGAAACTT	CGCAAAACCCACCCCATCATAAAAA	ACTTCGGATCACTACTAGGCATCTG
Tupaia belangeri	AACTTTGGATCCTTGCTAGGAATGT	CTTTGGATCCTTGCTAGGAATGTGC	TCATGGTGAAACTTTGGATCCTTGC
Urotrichus talpoides	TTCGGTTCACTATTAGGAATCTGCT	ACAAACCTACGAAAAACCCATCCCC	TCGATTTACCAGCACCCTCAAATAT
Ursus americanus	AACTTCGGATCTCTCCTCGGAGTAT	TATCTCAGCATGATGAAACTTCGGA	TCGGATCTCTCCTCGGAGTATGTTT
Ursus arctos	AATCCTACAGATTCTAACAGGCCTG	TCCTACAGATTCTAACAGGCCTGTT	CCTTCCAACACCATCAAACATCTCA
Ursus maritimus	GGATCCCTCCTTGGAGTGTGTTTAA	TTGGATCCCTCCTTGGAGTGTGTTT	GATCTTCCAACACCATCAAACATCT
Vombatus ursinus	CTCCAATATCTCCGCCTGATGAAAT	CGCCTGATGAAATTTTGGATCACTA	GACCTACCCACACCCTCCAATATCT

Probe list for microarray analysis using 150 bp of 5' region of COI in bats dataset

Species name	Probe 1	Probe 2	Probe 3
Ametrida centurio	TTATTCGTGCAGAACTTGGACAACC	ACTTGGACAACCTGGGGCTCTATTA	ATTCGTGCAGAACTTGGACAACCTG
Anoura caudifer	CAATGTAATCGTAACAGCCCATGCC	TACAATGTAATCGTAACAGCCCATG	ACAATGTAATCGTAACAGCCCATGC
Anoura geoffroyi	GTAACAGCTCATGCATTCGTGATAA	CGGTATAGTAGGCACTGCACTTAGC	GTATAGTAGGCACTGCACTTAGCCT
Anoura latidens	TATTATTTGGTGCCTGAGCTGGTAT	GGTATAGTAGGCACTGCACTTAGTC	GAGCTGGTATAGTAGGCACTGCACT
Artibeus amplus	No sequences		
Artibeus bogotensis	CACTGCATTAAGCCTTCTTATTCGT	AAGCCTTCTTATTCGTGCAGAACTT	AGTAGGCACTGCATTAAGCCTTCTT
Artibeus cinereus	TGCATTAAGCCTCCTTATTCGTGCA	GCATTAAGCCTCCTTATTCGTGCAG	CTCCTTATTCGTGCAGAACTTGGTC
Artibeus concolor	TATTATTCGGTGCTTGAGCAGGTAT	ATTATTCGGTGCTTGAGCAGGTATA	GTCTACTTATTCGTGCAGAACTTGG
Artibeus gnomus	GCCCTATTGGGTGACGACCAAATTT	TGTAATCGTAACAGCTCATGCTTTC	CCCTATTGGGTGACGACCAAATTTA
Artibeus lituratus	No sequences		
Artibeus obscurus	No sequences		
Artibeus planirostris	No sequences		
Carollia brevicauda PS1	No sequences		
Carollia brevicauda PS2	No sequences		
Carollia perspicillata	No sequences		
Chiroderma trinitatum	CCTAAGTCTCCTTATCCGTGCCGAA	ACCCTGTACTTATTATTTGGTGCCT	CTAAGTCTCCTTATCCGTGCCGAAC
Chiroderma villosum	ACTATTTGGTGCCTGAGCTGGTATA	TACTATTTGGTGCCTGAGCTGGTAT	CTATTTGGTGCCTGAGCTGGTATAG
Choeroniscus minor	GTAACAGCACATGCCTTCGTAATAA	CGGTATAGTAGGTACCGCACTTAGT	TCGTAACAGCACATGCCTTCGTAAT
Chrotopterus auritus	GCTTTACTGGGCGACGATCAAATCT	TGCTTTACTGGGCGACGATCAAATC	CATCGTAACAGCTCATGCCTTCGTA
Cormura brevirostris	CATTATACCTGCTATTCGGTGCTTG	ATTATACCTGCTATTCGGTGCTTGA	AATGTTATCGTTACTGCCCATGCAT
Cynomops paranus	TTCTCATTCGAGCTGAGTTAGGTCA	TTTACAACGTAATTGTCACCGCCCA	CCCTAAGTCTTCTCATTCGAGCTGA
Cynomops planirostris	CCAAATCTACAACGTAGTTGTCACC	CTTATCCGGGCTGAATTAGGTCAAC	GGTCAACCAGGAGCTCTTTTAGGAG
Cyttarops alecto	TGGAGACGACCAGATCTACAATGTA	GTAGTAGTCACTGCTCATGCATTTG	CTGCTTGGAGACGACCAGATCTACA
Desmodus rotundus	No sequences		
Diaemus youngi	CTACAACGTAGTAGTAACAGCCCAC	GACCCTTCTAGGTGATGACCAAATC	TATATCCTATTTGGAGCCTGAGCAG
Diclidurus isabellus	AGTCACAGCCCATGCTTTCGTAATA	ATAACGTAGTAGTCACAGCCCATGC	CTTGGGCAGGAATAGTAGGAACAGC
Eptesicus chiriquinus	GGAGCATTACTTGGGGATGATCAGA	TAATCCGTGCAGAATTAGGCCAACC	TACTAATCCGTGCAGAATTAGGCCA
Eptesicus furinalis	ATTCGTGCAGAATTAGGCCAACCAG	CTAATTCGTGCAGAATTAGGCCAAC	ACTAATTCGTGCAGAATTAGGCCAA
Eumops auripendulus	CCCTGAGTCTTTTAATCCGAGCTGA	TTAATCCGAGCTGAACTAGGACAGC	AATGTAATCGTCACAGCTCATGCCT
Eumops hansae	ATTTACAACGTAATCGTAACAGCCC	AATCGTAACAGCCCATGCTTTTGTT	TTTACAACGTAATCGTAACAGCCCA
Furipterus horrens	CTTTACATAATCTTCGGCGCTTGGG	GAGCCCTTATAGGCGACGATCAAAT	AGCCCTTATAGGCGACGATCAAATT
Glossophaga longirostris	GAGCTGGTATAGTAGGAACTGCATT	TGAGCTGGTATAGTAGGAACTGCAT	GCTCTATTGGGTGATGACCAGATTT
Glossophaga soricina	ATTAAGCCTACTTATCCGTGCCGAG	GAACCGCATTAAGCCTACTTATCCG	GGAACCGCATTAAGCCTACTTATCC
Glyphonycteris daviesi	ATTAAGTCTCCTTATTCGTGCCGAA	AAGTCTCCTTATTCGTGCCGAACTA	CTTATTCGTGCCGAACTAGGTCAGC
Glyphonycteris sylvestris	CTGTTGGGCGACGATCAAATCTATA	TCTATAACGTCGTCGTAACGGCTCA	GTATAGCAGGCACAGCTCTTAGTCT
Lampronycteris brachyotis	CCTTACTGGGGGACGATCAGATCTA	GGGACGATCAGATCTACAACGTTGT	TACTGGGGGACGATCAGATCTACAA
Lasiurus atratus	GTCACCGCTCATGCATTTGTAATGA	TTAGGCCAACCAGGTGCTCTTTTAG	GAGCCTACTTATTCGGGCTGAATTA
Lionycteris spurrelli	TATTATTTGGGGCTTGAGCAGGTAT	TTATTTGGGGCTTGAGCAGGTATAG	ATGTAATTGTGACAGCTCATGCCTT
Lonchophylla thomasi	GGTATAGTGGGTACTGCCCTTAGTC	ACTCTTTACTTACTGTTCGGAGCTT	TTTACTTACTGTTCGGAGCTTGAGC
Lonchorhina inusitata	GGGGCTTTACTAGGTGATGATCAAA	TAACCGCACACGCCTTTGTAATAAT	TGTAACCGCACACGCCTTTGTAATA
Lophostoma brasiliense	GAGCTCTTCTCGGTGATGACCAAAT	TAGTAACAGCTCATGCCTTCGTAAT	ACTAAGTCTTCTCATTCGTGCTGAG
Lophostoma carrikeri	ATGTTGTAGTAACAGCCCATGCCTT	TCTTCTTATTCGTGCTGAACTTGGA	TGTTGTAGTAACAGCCCATGCCTTC
Lophostoma schulzi	ATAACGTTGTAGTGACAGCACATGC	CATGGTAGGAACAGCATTAAGCCTT	GGCGATGACCAAATCTATAACGTTG
Lophostoma silvicolum	ATTTACAACGTTGTAGTAACAGCCC	GAGCTCTACTAGGCGATGACCAGAT	ACTAAGCCTCCTTATTCGTGCTGAA
Macrophyllum macrophyllum	AGCACTAAGTCTTCTCATTCGTGCC	ACTAAGTCTTCTCATTCGTGCCGAA	CTATACCTTATATTTGGCGCTTGGG
Mesophylla macconnelli	TATAGTGGGCACTGCCCTAAGTCTT	TGTAGTAGTAACAGCTCACGCCTTC	AATGTAGTAGTAACAGCTCACGCCT
Micronycteris hirsuta	TTTATATACTATTCGGCGCATGAGC	CATGAGCAGGTATAGTAGGAACTGC	CTGCTAGGGGATGACCAGATCTACA
Mimon crenulatum	GTTACAGCTCACGCTTTCGTAATAA	TTGTTACAGCTCACGCTTTCGTAAT	GTTATTGTTACAGCTCACGCTTTCG

Species name	Probe 1	Probe 2	Probe 3
Molossops neglectus	TTGGGAGACGACCAAATCTACAATG	GCTTTGAGTCTTCTTATCCGAGCCG	TCTTCTTATCCGAGCCGAATTAGGA
Molossus molossus	No sequences		
Molossus rufus	GTAATTGTAACCGCCCATGCCTTTG	TTGTAACCGCCCATGCCTTTGTAAT	TGTAATTGTAACCGCCCATGCCTTT
Molossus sp	CCTTCTTATCCGAGCTGAGCTAGGT	CTTCTTATCCGAGCTGAGCTAGGTC	TCTTATCCGAGCTGAGCTAGGTCAG
Myotis albescens	CACTGCATTAAGCTTACTAATCCGC	TAAGCTTACTAATCCGCGCCGAATT	AAGCTTACTAATCCGCGCCGAATTA
Myotis nigricans	TGCTTGAGCTGGAATAGTAGGCACT	ACCAGGAGCTCTACTAGGGGATGAT	ATAGTAGGCACTGCATTAAGCCTAC
Myotis riparius PS1	ATAGTAGGCACTGCATTGAGCTTAC	GTTACTGCTCACGCTTTTGTAATGA	TGTTACTGCTCACGCTTTTGTAATG
Myotis riparius PS2	GTAATTGTTACTGCTCACGCCTTTG	TTAAGCTTACTAATCCGCGCCGAAC	GTTACTGCTCACGCCTTTGTAATGA
Myotis riparius PS3	TAGGTCAACCAGGAGCTCTATTAGG	TCAACCAGGAGCTCTATTAGGGGAT	GTAGGTACTGCACTCAGCTTACTAA
Noctilio albiventris PS1	TTAGGTCAACCAGGCACTTTACTTG	CACTTTACTTGGCGATGACCAAATT	TAGGTCAACCAGGCACTTTACTTGG
Noctilio albiventris PS2	ATGTCGTAGTAACAGCCCATGCTTT	TTAGGTCAACCAGGTACTTTGCTTG	AGCAGAATTAGGTCAACCAGGTACT
Noctilio leporinus	GCACTTTGCTTGGTGATGACCAAAT	CCTAAGCCTAATCATCCGAGCAGAA	AATAGTAGGAACCGCCCTAAGCCTA
Nyctinomops macrotis	GTTACCGCTCACGCTTTTGTAATAA	CATCCGAGCTGAATTAGGTCAACCA	GCCTTCTCATCCGAGCTGAATTAGG
Peropteryx kappleri	GGTTACCGCCCACGCATTTGTAATA	GCCCTGCTAGGGGATGATCAAATTT	TATAATGTAGTGGTTACCGCCCACG
Peropteryx leucoptera	GTCACTGCCCATGCATTTGTAATAA	TACAATGTAGTGGTCACTGCCCATG	TGGTCACTGCCCATGCATTTGTAAT
Phylloderma stenops PS1	CTAGGAGACGACCAAATTTACAACG	TAGGAGACGACCAAATTTACAACGT	GACCAAATTTACAACGTCGTTGTCA
Phylloderma stenops PS2	TGAGCAGGACTAGTAGGTACTGCAC	TATACTTACTATTCGGTGCCTGAGC	GGCCCTACTAGGAGATGATCAAATT
Phyllostomus discolor	CGTTACGGCTCATGCTTTCGTAATG	TAGGAACTGCACTAAGCCTCCTCAT	TTACGGCTCATGCTTTCGTAATGAT
Phyllostomus elongatus	TCTATACCTCCTATTTGGTGCCTGA	GTATAGTGGGTACCGCACTTAGCCT	ACTCTATACCTCCTATTTGGTGCCT
Phyllostomus hastatus	TTATAATGTTGTCGTCACAGCCCAC	TTTATAATGTTGTCGTCACAGCCCA	CCCTCTTAGGGGATGACCAGATTTA
Platyrrhinus aurarius	GGCGATGACCAGATCTACAATGTAG	AGGCGATGACCAGATCTACAATGTA	GCGATGACCAGATCTACAATGTAGT
Platyrrhinus brachycephalus	GCCTTACTAGGCGATGATCAGATCT	TAGTAGTTACAGCTCATGCTTTCGT	CCCTATACTTGCTATTTGGTGCTTG
Platyrrhinus helleri PS1	GCTCTACTAGGTGATGACCAGATCT	TAAGCCTTCTTATCCGAGCTGAACT	GCTTGAGCTGGTATAGTTGGCACTG
Platyrrhinus helleri PS2	AATGTCGTAGTAACAGCTCATGCTT	ATGTCGTAGTAACAGCTCATGCTTT	CAATGTCGTAGTAACAGCTCATGCT
Platyrrhinus helleri PS3	GTCTCCTTATCCGAGCTGAACTTGG	CTCTAAGTCTCCTTATCCGAGCTGA	CACCGCTCTAAGTCTCCTTATCCGA
Pteronotus gymnonotus	TAGGGGATGACCAGATCTATAACGT	TCTACTTATCCGAGCAGAGCTAGGG	TATTTGGGGCCTGAGCAGGAATAGT
Pteronotus parnellii	ATAATGTTGTGGTTACAGCCCATGC	TGGTTACAGCCCATGCTTTCGTAAT	ATATCTACTATTCGGCGCCTGAGCA
Pteronotus personatus	ACAATGTAGTAGTCACAGCCCATGC	CTAGGTCAACCTGGAGCTCTGTTAG	TTTACAATGTAGTAGTCACAGCCCA
Rhinophylla pumilio	AATTAGGACAGCCAGGAGCATTACT	CTGAATTAGGACAGCCAGGAGCATT	TTATTCGTGCTGAATTAGGACAGCC
Rhynchonycteris naso	AGTTACAGCCCACGCATTTGTAATA	CGTAGTAGTTACAGCCCACGCATTT	AGCCGGAATAGTTGGTACAGCTCTA
Saccopteryx bilineata	ACAGCCCTAAGTCTCTTAATCCGTG	GCTTGAGCCGGAATAGTAGGTACAG	TTTATCTTCTATTTGGCGCTTGAGC
Saccopteryx canescens	TTTACCTCCTATTTGGTGCTTGAGC	GTAGTAGTCACTGCCCATGCATTCG	TACCTCCTATTTGGTGCTTGAGCAG
Saccopteryx gymnura	CTATAACGTAGTAGTTACCGCCCAT	TACAGCCCTAAGTCTCTTAATCCGC	TTACCTCCTATTCGGTGCTTGAGCA
Saccopteryx leptura	AGTCACCGCTCATGCATTCGTAATA	CACAGCCCTAAGCCTGTTAATTCGT	TTATCTTCTATTTGGTGCCTGAGCC
Sturnira lilium	TGAGCAGGAATAGTAGGAACTGCAC	AGCAGGAATAGTAGGAACTGCACTG	TTGAGCAGGAATAGTAGGAACTGCA
Sturnira tildae	ACGTTATCGTAACAGCCCATGCTTT	CAACGTTATCGTAACAGCCCATGCT	CGTTATCGTAACAGCCCATGCTTTC
Thyroptera tricolor	CTCATTATTCGTGCTGAACTCGGTC	ATTATTCGTGCTGAACTCGGTCAAC	TAAGCCTCATTATTCGTGCTGAACT
Tonatia saurophila	TAGGCACAGCACTAAGCCTCCTTAT	TCTATAATGTCATCGTTACAGCCCA	TAGGCCAACCAGGAGCACTATTAGG
Trachops cirrhosus PS1	CTGCACTAAGTCTCCTTATCCGTGC	CTATAATGTTGTTGTGACAGCCCAT	TATAATGTTGTTGTGACAGCCCATG
Trachops cirrhosus PS2	CTAAGTCTCCTCATTCGCGCTGAAC	GATCTATAACGTTGTTGTGACAGCT	TAACGTTGTTGTGACAGCTCATGCT
Trachops cirrhosus PS3	TTTACCTCTTATTTGGAGCCTGAGC	CCCTGCTAGGGGATGATCAGATCTA	TGTTGTAACAGCTCATGCTTTCGTA
Trinycteris nicefori	AGCAGGCATAGTAGGTACAGCATTA	GAGCAGGCATAGTAGGTACAGCATT	CTTTATCTACTGTTCGGTGCCTGAG
Uroderma bilobatum	No sequences		
Vampyressa bidens	TATCTACTATTCGGAGCTTGGGCTG	TACTATTCGGAGCTTGGGCTGGTAT	ACTATTCGGAGCTTGGGCTGGTATA
Vampyressa brocki	GTAACAGCCCATGCCTTCGTTATAA	AGCTTACTTATTCGCGCCGAACTAG	AAGCTTACTTATTCGCGCCGAACTA
Vampyressa thyone	CTTAAGCCTCCTCATTCGAGCTGAG	GAGCTGGTATAGTAGGCACTGCCTT	AGCTGGTATAGTAGGCACTGCCTTA
Vampyrodes caraccioli	ATAGTAGGTACTGCTTTGAGCCTCC	GCTCTACTAGGTGATGACCAAATCT	AGTAACAGCACACGCTTTCGTAATA
Vampyrum spectrum	AGGCGATGACCAGATCTACAATGTC	TACCGCATTGAGCCTTCTTATTCGC	CCCTATACCTATTGTTTGGGGCCTG
