# Supplementary material for: Design and applicability of DNA arrays and DNA barcodes in biodiversity monitoring
Source: BMC Biol. 2007 Jun 13;5:24. doi: 10.1186/1741-7007-5-24 (PMC1906742; doi:10.1186/1741-7007-5-24)
Supplement: Additional File 2 — Table of completely sequenced mammalian mitochondrial genomes used in our analyses as well as COI and cytb genes sequenced in this study. Shown are the species name, the common name, and the GenBank GI number for each sequence. [file 1741-7007-5-24-S2.pdf]

Additional file 2.

Table of completely sequenced mammalian mitochondrial genomes used in our analyses as well as COI and *cytb* genes sequenced in this study. Shown are the species name, the common name, and the GenBank ID number for each sequence.

Additional file 2
List of species and GenBank IDs

Species name	Common name	GenBank ID
Acinonyx jubatus	cheetah	38304374
Acinonyx jubatus	cheetah	38349475
Acinonyx jubatus	cheetah	38373498
Arctocephalus forsteri	New Zealand fur seal	21397128
Arctocephalus forsteri	New Zealand fur seal	21449916
Artibeus jamaicensis	Jamaican fruit-eating bat	4164474
Artibeus jamaicensis	Jamaican fruit-eating bat	5835666
Balaena mysticetus	bowhead whale	38707506
Balaena mysticetus	bowhead whale	62086650
Balaenoptera acutorostrata	minke whale	38707492
Balaenoptera bonaerensis	Antarctic minke whale	62086566
Balaenoptera bonaerensis	Antarctic minke whale	62086594
Balaenoptera bonaerensis	Antarctic minke whale	62184354
Balaenoptera borealis	sei whale	62086622
Balaenoptera borealis	sei whale	62184312
Balaenoptera brydei	Bryde's whale	62086608
Balaenoptera brydei	Bryde's whale	62184480
Balaenoptera brydei	Bryde's whale	90265648
Balaenoptera edeni	pygmy Bryde's whale	90265634
Balaenoptera edeni	pygmy Bryde's whale	91176188
Balaenoptera musculus	blue whale	414126
Balaenoptera musculus	blue whale	5834995
Balaenoptera omurai	Omura's baleen whale	90265606
Balaenoptera omurai	Omura's baleen whale	90265620
Balaenoptera omurai	Omura's baleen whale	91176262
Balaenoptera physalus	finback whale	12772
Balaenoptera physalus	finback whale	5819095
Berardius bairdii	Baird's beaked whale	38707598
Bos grunniens	yak	54654337
Bos grunniens	yak	55274615
Bos indicus	zebu cattle	33321647
Bos indicus	zebu cattle	37545825
Bos indicus	zebu cattle	50234066
Bos taurus	domestic cow	27543905
Bos taurus	domestic cow	27543919
Bos taurus	domestic cow	27543933
Bos taurus	domestic cow	27543947
Bos taurus	domestic cow	27543961
Bos taurus	domestic cow	27543975
Bos taurus	domestic cow	27543989
Bos taurus	domestic cow	33321661
Bos taurus	domestic cow	42521312
Bos taurus	domestic cow	56410894
Bos taurus	domestic cow	56410908
Bos taurus	domestic cow	56410922
Bos taurus	domestic cow	56410936
Bos taurus	domestic cow	56410950
Bos taurus	domestic cow	56410964
Bos taurus	domestic cow	56410978
Bos taurus	domestic cow	56410992
Bos taurus	domestic cow	56411006
Bos taurus	domestic cow	56411020
Bos taurus	domestic cow	56411034
Bos taurus	domestic cow	56411048
Bos taurus	domestic cow	56411062
Bos taurus	domestic cow	56411076

Bos taurus	domestic cow	56411090
Bos taurus	domestic cow	56411104
Bos taurus	domestic cow	56411118
Bos taurus	domestic cow	56411132
Bos taurus	domestic cow	56411146
Bos taurus	domestic cow	60101824
Bos taurus	domestic cow	70987468
Bos taurus	domestic cow	70987482
Bos taurus	domestic cow	70987496
Bos taurus	domestic cow	70987510
Bos taurus	domestic cow	70987524
Bos taurus	domestic cow	70987538
Bos taurus	domestic cow	70987552
Bos taurus	domestic cow	70987566
Bos taurus	domestic cow	70987580
Bos taurus	domestic cow	70987594
Bos taurus	domestic cow	70987608
Bos taurus	domestic cow	70987622
Bos taurus	domestic cow	70987636
Bos taurus	domestic cow	70987650
Bos taurus	domestic cow	70987664
Bos taurus	domestic cow	70987678
Bos taurus	domestic cow	70987692
Bos taurus	domestic cow	70987706
Bos taurus	domestic cow	70987720
Bos taurus	domestic cow	70987734
Bos taurus	domestic cow	70987748
Bos taurus	domestic cow	70987762
Bos taurus	domestic cow	70987776
Bos taurus	domestic cow	70987790
Bos taurus	domestic cow	70987804
Bos taurus	domestic cow	70987818
Bos taurus	domestic cow	70987832
Bos taurus	domestic cow	70987846
Bos taurus	domestic cow	70987860
Bos taurus	domestic cow	70987874
Bos taurus	domestic cow	70987888
Bos taurus	domestic cow	70987902
Bos taurus	domestic cow	70987916
Bos taurus	domestic cow	70987930
Bos taurus	domestic cow	70987944
Bos taurus	domestic cow	70987958
Bos taurus	domestic cow	70987972
Bos taurus	domestic cow	70987986
Bos taurus	domestic cow	70988000
Bos taurus	domestic cow	70988014
Bos taurus	domestic cow	70988028
Bos taurus	domestic cow	70988042
Bos taurus	domestic cow	70988056
Bos taurus	domestic cow	70988070
Bos taurus	domestic cow	70988084
Bos taurus	domestic cow	70988098
Bos taurus	domestic cow	70988112
Bos taurus	domestic cow	70988126
Bradypus tridactylus	pale-throated three-toed sloth	61660076
Bradypus tridactylus	pale-throated three-toed sloth	62184298
Bubalus bubalis	water buffalo	33334119
Bubalus bubalis	water buffalo	44894095
Bubalus bubalis	water buffalo	51173180

Bubalus bubalis	water buffalo	52220982
Caenolestes fuliginosus	silky shrew opossum	45685994
Canis familiaris	dogs	17737322
Canis familiaris	dogs	50301805
Canis familiaris	dogs	50301819
Canis familiaris	dogs	50301833
Canis familiaris	dogs	50301847
Canis familiaris	dogs	50301861
Canis familiaris	dogs	50301875
Canis familiaris	dogs	50301889
Canis familiaris	dogs	50301903
Canis familiaris	dogs	50301917
Canis familiaris	dogs	50301931
Canis familiaris	dogs	50301945
Canis familiaris	dogs	50301959
Canis familiaris	dogs	50301973
Canis familiaris	dogs	50301987
Canis familiaris	dogs	50302001
Canis familiaris	dogs	50302015
Canis familiaris	dogs	50302029
Canis familiaris	dogs	50302043
Canis familiaris	dogs	50302057
Canis familiaris	dogs	51872055
Canis familiaris	dogs	7534303
Canis familiaris	dogs	91702395
Canis familiaris	dogs	91702409
Canis familiaris	dogs	91702423
Canis familiaris	dogs	91702437
Canis familiaris	dogs	91702451
Canis familiaris	dogs	91702465
Canis familiaris	dogs	91702479
Canis familiaris	dogs	91702493
Canis familiaris	dogs	91702507
Canis familiaris	dogs	91702521
Canis familiaris	dogs	91702535
Canis familiaris	dogs	91702549
Canis familiaris	dogs	91702563
Canis familiaris	dogs	91702577
Canis latrans	coyote	108561416
Canis latrans	coyote	91702675
Canis latrans	coyote	91702689
Canis latrans	coyote	91702703
Canis lupus	gray wolf	108561299
Canis lupus	gray wolf	91702591
Canis lupus	gray wolf	91702605
Canis lupus	gray wolf	91702619
Canis lupus	gray wolf	91702633
Canis lupus	gray wolf	91702647
Canis lupus	gray wolf	91702661
Caperea marginata	pygmy right whale	38707520
Caperea marginata	pygmy right whale	62086692
Capra hircus	goats	33150920
Capra hircus	goats	33285125
Cavia porcellus	guinea pig	5835988
Cebus albifrons	white-fronted capuchin	14010679
Ceratotherium simum	white rhinoceros	5835401
Cervus elaphus	red deer	84095031
Cervus elaphus	red deer	84488580
Cervus nippon centralis	Honshu sika	62751070

Cervus nippon centralis	Honshu sika	63025126
Cervus nippon yakushimae	Yakushima sika deer	67625635
Cervus nippon yakushimae	Yakushima sika deer	68989228
Cervus nippon yesoensis	Hokkaido sika deer	62241577
Cervus nippon yesoensis	Hokkaido sika deer	62736193
Chalinolobus tuberculatus	New Zealand long-tailed bat	11545689
Chalinolobus tuberculatus	New Zealand long-tailed bat	11610804
Chlorocebus aethiops	vervet monkey	61743821
Chlorocebus aethiops	vervet monkey	66276030
Chlorocebus sabaeus	green monkey	107736076
Chlorocebus sabaeus	green monkey	67082892
Choloepus didactylus	southern two-toed sloth	61660090
Choloepus didactylus	southern two-toed sloth	62184340
Chrysochloris asiatica	N/A	32187994
Chrysochloris asiatica	N/A	32402539
Colobus guereza	guereza	60392100
Colobus guereza	guereza	62161140
Cricetulus griseus	striped dwarf hamster	89258123
Cricetulus griseus	striped dwarf hamster	91176202
Crocidura russula	white-toothed shrew	54397753
Crocidura russula	white-toothed shrew	54397767
Crocidura russula	white-toothed shrew	62161165
Cynocephalus variegatus	Malayan flying lemur	21449889
Cynocephalus variegatus	Malayan flying lemur	26284395
Dactylopsila trivirgata	striped possum	108793294
Dactylopsila trivirgata	striped possum	94481173
Dasypus novemcinctus	nine-banded armadillo	5835429
Dasyurus hallucatus	northern quoll	55416188
Dasyurus hallucatus	northern quoll	83309015
Didelphis virginiana	Virginia opossum	5835037
Distoechurus pennatus	feathertail possum	108793280
Distoechurus pennatus	feathertail possum	94481145
Dromiciops gliroides	monito del monte	45685691
Dugong dugon	dugong	17981678
Dugong dugon	dugong	18913043
Echinops telfairi	small Madagascar hedgehog	32469193
Echinops telfairi	small Madagascar hedgehog	34582602
Echinosorex gymnura	N/A	14495261
Echinosorex gymnura	N/A	14530815
Echymipera rufescens australis	N/A	55416216
Echymipera rufescens australis	N/A	83309085
Elephantulus sp. VB001	N/A	32188008
Elephantulus sp. VB001	N/A	32402553
Elephas maximus	Indian elephant	107122072
Elephas maximus	Indian elephant	83415390
Episoriculus fumidus	Taiwan brown-toothed shrew	14599777
Episoriculus fumidus	Taiwan brown-toothed shrew	15079203
Equus asinus	donkey	5835345
Equus caballus	horse	47156680
Equus caballus	horse	5835107
Erinaceus europaeus	western European hedgehog	5835792
Eschrichtius robustus	grey whale	38707478
Eschrichtius robustus	grey whale	62086636
Eubalaena australis	Southern right whale	62086664
Eubalaena australis	Southern right whale	62184410
Eubalaena japonica	North Pacific right whale	62086678
Eubalaena japonica	North Pacific right whale	62184536
Eumetopias jubatus	northern sea lion	34577124
Felis catus	domestic cat	1098523

Felis catus	domestic cat	5835205
Galemys pyrenaicus	N/A	109156429
Galemys pyrenaicus	N/A	56714039
Gorilla gorilla	gorilla	5835149
Gorilla gorilla gorilla	lowland gorilla	1304307
Halichoerus grypus	grey seal	5835009
Hemiechinus auritus	long-eared hedgehog	32469151
Hemiechinus auritus	long-eared hedgehog	32526842
Herpestes javanicus	N/A	57014054
Herpestes javanicus	N/A	58578650
Hippopotamus amphibius	hippopotamus	5836030
Homo sapiens	man	107784936
Homo sapiens	man	107785150
Homo sapiens	man	109255141
Homo sapiens	man	109255143
Homo sapiens	man	109692789
Homo sapiens	man	109893899
Homo sapiens	man	109894756
Homo sapiens	man	110083928
Homo sapiens	man	110169664
Homo sapiens	man	110169678
Homo sapiens	man	110169692
Homo sapiens	man	110169706
Homo sapiens	man	110169720
Homo sapiens	man	110169734
Homo sapiens	man	110169748
Homo sapiens	man	110169762
Homo sapiens	man	110169776
Homo sapiens	man	110351027
Homo sapiens	man	110609912
Homo sapiens	man	110609913
Homo sapiens	man	110609914
Homo sapiens	man	110609915
Homo sapiens	man	110609916
Homo sapiens	man	110609917
Homo sapiens	man	110609918
Homo sapiens	man	110609919
Homo sapiens	man	110609920
Homo sapiens	man	110609921
Homo sapiens	man	110609922
Homo sapiens	man	110609923
Homo sapiens	man	110609924
Homo sapiens	man	110609925
Homo sapiens	man	110609926
Homo sapiens	man	110609927
Homo sapiens	man	110609928
Homo sapiens	man	110609929
Homo sapiens	man	110609930
Homo sapiens	man	110609931
Homo sapiens	man	110609932
Homo sapiens	man	110609933
Homo sapiens	man	110609934
Homo sapiens	man	110609935
Homo sapiens	man	110609936
Homo sapiens	man	110609937
Homo sapiens	man	110609938
Homo sapiens	man	110609939
Homo sapiens	man	110609940
Homo sapiens	man	110609941

Homo sapiens	man	110609942
Homo sapiens	man	110609943
Homo sapiens	man	110609944
Homo sapiens	man	110609945
Homo sapiens	man	110609946
Homo sapiens	man	110609947
Homo sapiens	man	110609948
Homo sapiens	man	110609949
Homo sapiens	man	110609950
Homo sapiens	man	110609951
Homo sapiens	man	110609952
Homo sapiens	man	110609953
Homo sapiens	man	110609954
Homo sapiens	man	110609955
Homo sapiens	man	110609956
Homo sapiens	man	110609957
Homo sapiens	man	110609958
Homo sapiens	man	110609960
Homo sapiens	man	110609962
Homo sapiens	man	110609963
Homo sapiens	man	110609964
Homo sapiens	man	110609965
Homo sapiens	man	110609966
Homo sapiens	man	110609967
Homo sapiens	man	110609968
Homo sapiens	man	110609969
Homo sapiens	man	110609970
Homo sapiens	man	110609971
Homo sapiens	man	110609972
Homo sapiens	man	110609973
Homo sapiens	man	110609974
Homo sapiens	man	110809874
Homo sapiens	man	110809888
Homo sapiens	man	111218090
Homo sapiens	man	111218091
Homo sapiens	man	113200490
Homo sapiens	man	113201060
Homo sapiens	man	113201074
Homo sapiens	man	113201088
Homo sapiens	man	113201102
Homo sapiens	man	113201116
Homo sapiens	man	113201130
Homo sapiens	man	113201144
Homo sapiens	man	113201158
Homo sapiens	man	113201172
Homo sapiens	man	113201186
Homo sapiens	man	113201200
Homo sapiens	man	113201214
Homo sapiens	man	113201228
Homo sapiens	man	113201242
Homo sapiens	man	113201256
Homo sapiens	man	113201270
Homo sapiens	man	113201284
Homo sapiens	man	113201298
Homo sapiens	man	113201540
Homo sapiens	man	113201554
Homo sapiens	man	113201568
Homo sapiens	man	113201582
Homo sapiens	man	113201596

Homo sapiens	man	113201610
Homo sapiens	man	113201624
Homo sapiens	man	113201638
Homo sapiens	man	113201652
Homo sapiens	man	113706986
Homo sapiens	man	113706988
Homo sapiens	man	113706990
Homo sapiens	man	113706992
Homo sapiens	man	113706994
Homo sapiens	man	113706996
Homo sapiens	man	113706998
Homo sapiens	man	113707000
Homo sapiens	man	1262342
Homo sapiens	man	13272556
Homo sapiens	man	13272570
Homo sapiens	man	13272584
Homo sapiens	man	13272598
Homo sapiens	man	13272612
Homo sapiens	man	13272626
Homo sapiens	man	13272640
Homo sapiens	man	13272654
Homo sapiens	man	13272668
Homo sapiens	man	13272682
Homo sapiens	man	13272696
Homo sapiens	man	13272710
Homo sapiens	man	13272724
Homo sapiens	man	13272738
Homo sapiens	man	13272752
Homo sapiens	man	13272766
Homo sapiens	man	13272780
Homo sapiens	man	13272794
Homo sapiens	man	13272808
Homo sapiens	man	13272822
Homo sapiens	man	13272836
Homo sapiens	man	13272850
Homo sapiens	man	13272864
Homo sapiens	man	13272878
Homo sapiens	man	13272892
Homo sapiens	man	13272906
Homo sapiens	man	13272920
Homo sapiens	man	13272934
Homo sapiens	man	13272948
Homo sapiens	man	13272962
Homo sapiens	man	13272976
Homo sapiens	man	13272990
Homo sapiens	man	13273004
Homo sapiens	man	13273018
Homo sapiens	man	13273032
Homo sapiens	man	13273046
Homo sapiens	man	13273060
Homo sapiens	man	13273074
Homo sapiens	man	13273088
Homo sapiens	man	13273102
Homo sapiens	man	13273116
Homo sapiens	man	13273130
Homo sapiens	man	13273144
Homo sapiens	man	13273158
Homo sapiens	man	13273172
Homo sapiens	man	13273186

Homo sapiens	man	13273200
Homo sapiens	man	13273214
Homo sapiens	man	13273228
Homo sapiens	man	13273242
Homo sapiens	man	13273256
Homo sapiens	man	13273270
Homo sapiens	man	13273284
Homo sapiens	man	17981852
Homo sapiens	man	17985375
Homo sapiens	man	17985389
Homo sapiens	man	17985403
Homo sapiens	man	17985431
Homo sapiens	man	17985445
Homo sapiens	man	17985459
Homo sapiens	man	17985473
Homo sapiens	man	17985487
Homo sapiens	man	17985501
Homo sapiens	man	17985515
Homo sapiens	man	17985543
Homo sapiens	man	17985571
Homo sapiens	man	17985599
Homo sapiens	man	17985613
Homo sapiens	man	17985627
Homo sapiens	man	17985641
Homo sapiens	man	17985655
Homo sapiens	man	17985669
Homo sapiens	man	17985683
Homo sapiens	man	17985697
Homo sapiens	man	17985711
Homo sapiens	man	17985725
Homo sapiens	man	17985739
Homo sapiens	man	17985781
Homo sapiens	man	17985795
Homo sapiens	man	17985809
Homo sapiens	man	17985823
Homo sapiens	man	29294531
Homo sapiens	man	29294532
Homo sapiens	man	29294533
Homo sapiens	man	29294534
Homo sapiens	man	29690434
Homo sapiens	man	29690462
Homo sapiens	man	29690476
Homo sapiens	man	29690560
Homo sapiens	man	29690616
Homo sapiens	man	29690658
Homo sapiens	man	29690686
Homo sapiens	man	29690756
Homo sapiens	man	29690784
Homo sapiens	man	29690840
Homo sapiens	man	29690882
Homo sapiens	man	29690980
Homo sapiens	man	32347988
Homo sapiens	man	32348002
Homo sapiens	man	32348016
Homo sapiens	man	32348030
Homo sapiens	man	32348044
Homo sapiens	man	32348058
Homo sapiens	man	32348072
Homo sapiens	man	32348086

Homo sapiens	man	32348100
Homo sapiens	man	32348114
Homo sapiens	man	32348128
Homo sapiens	man	32348142
Homo sapiens	man	32348156
Homo sapiens	man	32348170
Homo sapiens	man	32348184
Homo sapiens	man	32348198
Homo sapiens	man	32348212
Homo sapiens	man	32348226
Homo sapiens	man	32348240
Homo sapiens	man	32348254
Homo sapiens	man	32348268
Homo sapiens	man	32348282
Homo sapiens	man	32348296
Homo sapiens	man	32348310
Homo sapiens	man	32348324
Homo sapiens	man	32348338
Homo sapiens	man	32348352
Homo sapiens	man	32348366
Homo sapiens	man	32348380
Homo sapiens	man	32348394
Homo sapiens	man	32348408
Homo sapiens	man	32348422
Homo sapiens	man	32348436
Homo sapiens	man	32348450
Homo sapiens	man	32348464
Homo sapiens	man	32348478
Homo sapiens	man	32348492
Homo sapiens	man	32348506
Homo sapiens	man	32348520
Homo sapiens	man	32348534
Homo sapiens	man	32348548
Homo sapiens	man	32348562
Homo sapiens	man	32348575
Homo sapiens	man	32348589
Homo sapiens	man	32348603
Homo sapiens	man	32348617
Homo sapiens	man	32348631
Homo sapiens	man	32348645
Homo sapiens	man	32348659
Homo sapiens	man	32348673
Homo sapiens	man	32348687
Homo sapiens	man	32348701
Homo sapiens	man	32891089
Homo sapiens	man	32891117
Homo sapiens	man	32891145
Homo sapiens	man	32891159
Homo sapiens	man	32891173
Homo sapiens	man	32891187
Homo sapiens	man	32891201
Homo sapiens	man	32891215
Homo sapiens	man	32891229
Homo sapiens	man	32891243
Homo sapiens	man	32891257
Homo sapiens	man	32891271
Homo sapiens	man	32891285
Homo sapiens	man	32891299
Homo sapiens	man	32891313

Homo sapiens	man	32891327
Homo sapiens	man	32891341
Homo sapiens	man	32891355
Homo sapiens	man	32891369
Homo sapiens	man	32891383
Homo sapiens	man	32891397
Homo sapiens	man	32891411
Homo sapiens	man	32891425
Homo sapiens	man	32891439
Homo sapiens	man	32891453
Homo sapiens	man	32891467
Homo sapiens	man	32891481
Homo sapiens	man	32891495
Homo sapiens	man	32891509
Homo sapiens	man	32891523
Homo sapiens	man	32891537
Homo sapiens	man	32891551
Homo sapiens	man	32891565
Homo sapiens	man	32891579
Homo sapiens	man	32891592
Homo sapiens	man	32891606
Homo sapiens	man	32891620
Homo sapiens	man	32891634
Homo sapiens	man	32891648
Homo sapiens	man	32891662
Homo sapiens	man	32891676
Homo sapiens	man	32891690
Homo sapiens	man	32891704
Homo sapiens	man	32891732
Homo sapiens	man	32891746
Homo sapiens	man	32892309
Homo sapiens	man	32892323
Homo sapiens	man	32892337
Homo sapiens	man	32892351
Homo sapiens	man	32892365
Homo sapiens	man	32892379
Homo sapiens	man	32892393
Homo sapiens	man	32892435
Homo sapiens	man	32892449
Homo sapiens	man	32892463
Homo sapiens	man	32892477
Homo sapiens	man	32892491
Homo sapiens	man	32892505
Homo sapiens	man	32892519
Homo sapiens	man	32892533
Homo sapiens	man	32892547
Homo sapiens	man	32892561
Homo sapiens	man	32892575
Homo sapiens	man	32892589
Homo sapiens	man	32892603
Homo sapiens	man	32892617
Homo sapiens	man	32892631
Homo sapiens	man	32892645
Homo sapiens	man	32892659
Homo sapiens	man	32892673
Homo sapiens	man	32892687
Homo sapiens	man	32892701
Homo sapiens	man	32892715
Homo sapiens	man	32892729

Homo sapiens	man	32892743
Homo sapiens	man	32892757
Homo sapiens	man	32892771
Homo sapiens	man	32892785
Homo sapiens	man	32892799
Homo sapiens	man	32892813
Homo sapiens	man	32892827
Homo sapiens	man	32892841
Homo sapiens	man	32892855
Homo sapiens	man	32892869
Homo sapiens	man	32892883
Homo sapiens	man	32892897
Homo sapiens	man	32892911
Homo sapiens	man	32892925
Homo sapiens	man	32892939
Homo sapiens	man	32892953
Homo sapiens	man	32892967
Homo sapiens	man	32892981
Homo sapiens	man	32892995
Homo sapiens	man	32893009
Homo sapiens	man	32893023
Homo sapiens	man	32893037
Homo sapiens	man	32893051
Homo sapiens	man	32893065
Homo sapiens	man	32893079
Homo sapiens	man	32893093
Homo sapiens	man	32893107
Homo sapiens	man	32893275
Homo sapiens	man	32893653
Homo sapiens	man	32893667
Homo sapiens	man	32893681
Homo sapiens	man	32893695
Homo sapiens	man	32893709
Homo sapiens	man	32893751
Homo sapiens	man	32893877
Homo sapiens	man	32893891
Homo sapiens	man	32893905
Homo sapiens	man	32893919
Homo sapiens	man	32893933
Homo sapiens	man	32893947
Homo sapiens	man	32893961
Homo sapiens	man	32893975
Homo sapiens	man	32893989
Homo sapiens	man	32894003
Homo sapiens	man	32894017
Homo sapiens	man	32894031
Homo sapiens	man	32894045
Homo sapiens	man	32894059
Homo sapiens	man	32894073
Homo sapiens	man	32894101
Homo sapiens	man	32894115
Homo sapiens	man	32894129
Homo sapiens	man	32894143
Homo sapiens	man	32894157
Homo sapiens	man	32894171
Homo sapiens	man	32894185
Homo sapiens	man	32894199
Homo sapiens	man	32894213
Homo sapiens	man	32894227

Homo sapiens	man	32894241
Homo sapiens	man	32894255
Homo sapiens	man	32894269
Homo sapiens	man	32894283
Homo sapiens	man	32894297
Homo sapiens	man	32894311
Homo sapiens	man	32894325
Homo sapiens	man	32894339
Homo sapiens	man	32894353
Homo sapiens	man	32894367
Homo sapiens	man	32894381
Homo sapiens	man	32894395
Homo sapiens	man	32894409
Homo sapiens	man	32894423
Homo sapiens	man	32894605
Homo sapiens	man	32894619
Homo sapiens	man	32894633
Homo sapiens	man	32894647
Homo sapiens	man	32894661
Homo sapiens	man	32894675
Homo sapiens	man	32894689
Homo sapiens	man	32894703
Homo sapiens	man	32894717
Homo sapiens	man	32894731
Homo sapiens	man	32894745
Homo sapiens	man	32894759
Homo sapiens	man	32894773
Homo sapiens	man	32894787
Homo sapiens	man	32894801
Homo sapiens	man	32894829
Homo sapiens	man	32894843
Homo sapiens	man	32894857
Homo sapiens	man	32894871
Homo sapiens	man	32894885
Homo sapiens	man	32894899
Homo sapiens	man	32894913
Homo sapiens	man	32894927
Homo sapiens	man	32894941
Homo sapiens	man	32894955
Homo sapiens	man	32894969
Homo sapiens	man	32894983
Homo sapiens	man	33465897
Homo sapiens	man	33465911
Homo sapiens	man	33465925
Homo sapiens	man	33465939
Homo sapiens	man	33465953
Homo sapiens	man	33465967
Homo sapiens	man	33465981
Homo sapiens	man	33465995
Homo sapiens	man	33466009
Homo sapiens	man	33466023
Homo sapiens	man	33466037
Homo sapiens	man	37954121
Homo sapiens	man	40795190
Homo sapiens	man	40795218
Homo sapiens	man	40846459
Homo sapiens	man	40846473
Homo sapiens	man	40846487
Homo sapiens	man	40846501

Homo sapiens	man	40846515
Homo sapiens	man	40846529
Homo sapiens	man	40846543
Homo sapiens	man	40846557
Homo sapiens	man	40846571
Homo sapiens	man	40846585
Homo sapiens	man	40846599
Homo sapiens	man	40846613
Homo sapiens	man	40846627
Homo sapiens	man	40846641
Homo sapiens	man	40846655
Homo sapiens	man	40846697
Homo sapiens	man	40846711
Homo sapiens	man	40846725
Homo sapiens	man	40846739
Homo sapiens	man	40846753
Homo sapiens	man	40846767
Homo sapiens	man	40846781
Homo sapiens	man	40846795
Homo sapiens	man	40846809
Homo sapiens	man	40846823
Homo sapiens	man	40846837
Homo sapiens	man	40846851
Homo sapiens	man	40846865
Homo sapiens	man	40846879
Homo sapiens	man	40846893
Homo sapiens	man	40846907
Homo sapiens	man	40846935
Homo sapiens	man	40846949
Homo sapiens	man	40846963
Homo sapiens	man	40847005
Homo sapiens	man	40847019
Homo sapiens	man	40847033
Homo sapiens	man	40847047
Homo sapiens	man	40847061
Homo sapiens	man	40847075
Homo sapiens	man	40847089
Homo sapiens	man	40847103
Homo sapiens	man	40847117
Homo sapiens	man	40847131
Homo sapiens	man	40847145
Homo sapiens	man	40847159
Homo sapiens	man	40847173
Homo sapiens	man	40847187
Homo sapiens	man	40847215
Homo sapiens	man	40847243
Homo sapiens	man	40847257
Homo sapiens	man	40847271
Homo sapiens	man	40847285
Homo sapiens	man	40847299
Homo sapiens	man	40847313
Homo sapiens	man	40847327
Homo sapiens	man	40847341
Homo sapiens	man	40847355
Homo sapiens	man	40847369
Homo sapiens	man	40847397
Homo sapiens	man	40847411
Homo sapiens	man	40847425
Homo sapiens	man	40847439

Homo sapiens	man	40847453
Homo sapiens	man	40847467
Homo sapiens	man	40847481
Homo sapiens	man	40847495
Homo sapiens	man	40847509
Homo sapiens	man	40847523
Homo sapiens	man	40847537
Homo sapiens	man	40847551
Homo sapiens	man	40847565
Homo sapiens	man	40847579
Homo sapiens	man	40847593
Homo sapiens	man	40847607
Homo sapiens	man	40847621
Homo sapiens	man	40847635
Homo sapiens	man	40847649
Homo sapiens	man	40847663
Homo sapiens	man	40847677
Homo sapiens	man	40847691
Homo sapiens	man	40847705
Homo sapiens	man	40847719
Homo sapiens	man	40847733
Homo sapiens	man	40847747
Homo sapiens	man	40847761
Homo sapiens	man	40847775
Homo sapiens	man	40847789
Homo sapiens	man	40847803
Homo sapiens	man	40847817
Homo sapiens	man	40847831
Homo sapiens	man	40847845
Homo sapiens	man	40847859
Homo sapiens	man	40847873
Homo sapiens	man	40847887
Homo sapiens	man	40847901
Homo sapiens	man	40847915
Homo sapiens	man	40847929
Homo sapiens	man	40847943
Homo sapiens	man	40847957
Homo sapiens	man	40847971
Homo sapiens	man	40847985
Homo sapiens	man	40847999
Homo sapiens	man	40848013
Homo sapiens	man	40848027
Homo sapiens	man	40848041
Homo sapiens	man	40848055
Homo sapiens	man	40848069
Homo sapiens	man	40848083
Homo sapiens	man	40848097
Homo sapiens	man	40848111
Homo sapiens	man	40848125
Homo sapiens	man	40848139
Homo sapiens	man	40848153
Homo sapiens	man	40848167
Homo sapiens	man	40848181
Homo sapiens	man	40848195
Homo sapiens	man	40848209
Homo sapiens	man	40848223
Homo sapiens	man	40848237
Homo sapiens	man	40848251
Homo sapiens	man	40848265

Homo sapiens	man	40848279
Homo sapiens	man	40848293
Homo sapiens	man	40848307
Homo sapiens	man	40848321
Homo sapiens	man	40848335
Homo sapiens	man	40848349
Homo sapiens	man	40848363
Homo sapiens	man	40848377
Homo sapiens	man	40848391
Homo sapiens	man	40848405
Homo sapiens	man	40848419
Homo sapiens	man	40848433
Homo sapiens	man	40848447
Homo sapiens	man	40848461
Homo sapiens	man	40848475
Homo sapiens	man	40848489
Homo sapiens	man	40848503
Homo sapiens	man	40848517
Homo sapiens	man	40848531
Homo sapiens	man	40848545
Homo sapiens	man	40848559
Homo sapiens	man	40848573
Homo sapiens	man	40848587
Homo sapiens	man	40848601
Homo sapiens	man	40848615
Homo sapiens	man	40848629
Homo sapiens	man	40848643
Homo sapiens	man	40848657
Homo sapiens	man	40848671
Homo sapiens	man	40848685
Homo sapiens	man	40848699
Homo sapiens	man	40848713
Homo sapiens	man	40848727
Homo sapiens	man	40848741
Homo sapiens	man	40848755
Homo sapiens	man	40848769
Homo sapiens	man	40848783
Homo sapiens	man	40848797
Homo sapiens	man	40848811
Homo sapiens	man	40848825
Homo sapiens	man	40848839
Homo sapiens	man	40848853
Homo sapiens	man	40848867
Homo sapiens	man	40848881
Homo sapiens	man	40848895
Homo sapiens	man	40848909
Homo sapiens	man	40848923
Homo sapiens	man	40848937
Homo sapiens	man	40848951
Homo sapiens	man	40848965
Homo sapiens	man	40848979
Homo sapiens	man	40848993
Homo sapiens	man	40849007
Homo sapiens	man	40849021
Homo sapiens	man	40849035
Homo sapiens	man	40849049
Homo sapiens	man	40849063
Homo sapiens	man	40849077
Homo sapiens	man	40849091

Homo sapiens	man	40849105
Homo sapiens	man	40849119
Homo sapiens	man	40849133
Homo sapiens	man	40849147
Homo sapiens	man	40849161
Homo sapiens	man	40849175
Homo sapiens	man	40849189
Homo sapiens	man	40849203
Homo sapiens	man	40849217
Homo sapiens	man	40849231
Homo sapiens	man	40849245
Homo sapiens	man	40849259
Homo sapiens	man	40849273
Homo sapiens	man	40849287
Homo sapiens	man	40849301
Homo sapiens	man	40849315
Homo sapiens	man	40849329
Homo sapiens	man	40849343
Homo sapiens	man	40849357
Homo sapiens	man	40849371
Homo sapiens	man	40849385
Homo sapiens	man	40849399
Homo sapiens	man	40849413
Homo sapiens	man	40849427
Homo sapiens	man	40849441
Homo sapiens	man	40849455
Homo sapiens	man	40849469
Homo sapiens	man	40849483
Homo sapiens	man	40849497
Homo sapiens	man	40849511
Homo sapiens	man	40849525
Homo sapiens	man	40849539
Homo sapiens	man	40849553
Homo sapiens	man	40849567
Homo sapiens	man	40849581
Homo sapiens	man	40849595
Homo sapiens	man	40849609
Homo sapiens	man	40849623
Homo sapiens	man	40849637
Homo sapiens	man	40849651
Homo sapiens	man	40849665
Homo sapiens	man	40849679
Homo sapiens	man	40849693
Homo sapiens	man	40849707
Homo sapiens	man	40849721
Homo sapiens	man	40849735
Homo sapiens	man	40849749
Homo sapiens	man	40849763
Homo sapiens	man	40849777
Homo sapiens	man	40849791
Homo sapiens	man	40849805
Homo sapiens	man	40849819
Homo sapiens	man	46242454
Homo sapiens	man	46242482
Homo sapiens	man	47717682
Homo sapiens	man	47717710
Homo sapiens	man	48131404
Homo sapiens	man	48526882
Homo sapiens	man	48526883

Homo sapiens	man	48596135
Homo sapiens	man	48596136
Homo sapiens	man	48596138
Homo sapiens	man	48596141
Homo sapiens	man	48596143
Homo sapiens	man	48596144
Homo sapiens	man	48596151
Homo sapiens	man	48596153
Homo sapiens	man	48596154
Homo sapiens	man	48596155
Homo sapiens	man	48596156
Homo sapiens	man	48596157
Homo sapiens	man	48596164
Homo sapiens	man	48596168
Homo sapiens	man	48596173
Homo sapiens	man	48596175
Homo sapiens	man	48596176
Homo sapiens	man	48596177
Homo sapiens	man	48596178
Homo sapiens	man	48596180
Homo sapiens	man	48596189
Homo sapiens	man	48596190
Homo sapiens	man	48596191
Homo sapiens	man	48596193
Homo sapiens	man	48596195
Homo sapiens	man	48596196
Homo sapiens	man	48596197
Homo sapiens	man	48596203
Homo sapiens	man	48596205
Homo sapiens	man	48596208
Homo sapiens	man	48596214
Homo sapiens	man	48596215
Homo sapiens	man	48596217
Homo sapiens	man	48596219
Homo sapiens	man	48596221
Homo sapiens	man	48596223
Homo sapiens	man	49472657
Homo sapiens	man	50295411
Homo sapiens	man	50295412
Homo sapiens	man	50295413
Homo sapiens	man	50295414
Homo sapiens	man	50295415
Homo sapiens	man	50295416
Homo sapiens	man	50295417
Homo sapiens	man	50295419
Homo sapiens	man	50295420
Homo sapiens	man	50295421
Homo sapiens	man	50295422
Homo sapiens	man	50295423
Homo sapiens	man	50295424
Homo sapiens	man	50295425
Homo sapiens	man	51450294
Homo sapiens	man	51450308
Homo sapiens	man	51450322
Homo sapiens	man	51450336
Homo sapiens	man	51450350
Homo sapiens	man	51450364
Homo sapiens	man	51450378
Homo sapiens	man	51450392

Homo sapiens	man	51450406
Homo sapiens	man	51450420
Homo sapiens	man	51450434
Homo sapiens	man	51450448
Homo sapiens	man	51450462
Homo sapiens	man	51450476
Homo sapiens	man	51450490
Homo sapiens	man	51450504
Homo sapiens	man	51450518
Homo sapiens	man	51450532
Homo sapiens	man	51450546
Homo sapiens	man	51450560
Homo sapiens	man	51450574
Homo sapiens	man	51450588
Homo sapiens	man	51450602
Homo sapiens	man	51450616
Homo sapiens	man	51450630
Homo sapiens	man	51450644
Homo sapiens	man	51450658
Homo sapiens	man	51450672
Homo sapiens	man	51450686
Homo sapiens	man	51450700
Homo sapiens	man	51450714
Homo sapiens	man	51450728
Homo sapiens	man	51450742
Homo sapiens	man	51450756
Homo sapiens	man	51450770
Homo sapiens	man	51450784
Homo sapiens	man	51450798
Homo sapiens	man	51450812
Homo sapiens	man	51450826
Homo sapiens	man	51450840
Homo sapiens	man	51450854
Homo sapiens	man	51450868
Homo sapiens	man	51450882
Homo sapiens	man	51450896
Homo sapiens	man	51450910
Homo sapiens	man	51450924
Homo sapiens	man	51450938
Homo sapiens	man	51450952
Homo sapiens	man	51450966
Homo sapiens	man	51450980
Homo sapiens	man	51450994
Homo sapiens	man	51451008
Homo sapiens	man	51451022
Homo sapiens	man	51451036
Homo sapiens	man	51451050
Homo sapiens	man	51451064
Homo sapiens	man	51451078
Homo sapiens	man	51451092
Homo sapiens	man	51451106
Homo sapiens	man	51451120
Homo sapiens	man	51451134
Homo sapiens	man	51451148
Homo sapiens	man	51451162
Homo sapiens	man	51451176
Homo sapiens	man	51451190
Homo sapiens	man	51451204
Homo sapiens	man	51451218

Homo sapiens	man	51451232
Homo sapiens	man	51451246
Homo sapiens	man	51451260
Homo sapiens	man	51451274
Homo sapiens	man	51451288
Homo sapiens	man	51451302
Homo sapiens	man	51451316
Homo sapiens	man	51451330
Homo sapiens	man	51894476
Homo sapiens	man	51894490
Homo sapiens	man	51894504
Homo sapiens	man	51894518
Homo sapiens	man	51894532
Homo sapiens	man	51894546
Homo sapiens	man	51894560
Homo sapiens	man	51894574
Homo sapiens	man	51894588
Homo sapiens	man	51894602
Homo sapiens	man	51894616
Homo sapiens	man	51894630
Homo sapiens	man	51894644
Homo sapiens	man	51894658
Homo sapiens	man	51894672
Homo sapiens	man	51894686
Homo sapiens	man	51894700
Homo sapiens	man	51894714
Homo sapiens	man	51894728
Homo sapiens	man	51894742
Homo sapiens	man	51894756
Homo sapiens	man	51894770
Homo sapiens	man	51894784
Homo sapiens	man	51894798
Homo sapiens	man	51894812
Homo sapiens	man	51894826
Homo sapiens	man	51894840
Homo sapiens	man	51894854
Homo sapiens	man	51894868
Homo sapiens	man	51894882
Homo sapiens	man	51894896
Homo sapiens	man	51894910
Homo sapiens	man	51894924
Homo sapiens	man	51894938
Homo sapiens	man	51894952
Homo sapiens	man	51894966
Homo sapiens	man	51894980
Homo sapiens	man	51894994
Homo sapiens	man	51895008
Homo sapiens	man	51895022
Homo sapiens	man	51895036
Homo sapiens	man	51895050
Homo sapiens	man	51895064
Homo sapiens	man	51895078
Homo sapiens	man	51895092
Homo sapiens	man	51895106
Homo sapiens	man	51895120
Homo sapiens	man	51895134
Homo sapiens	man	51895148
Homo sapiens	man	51895162
Homo sapiens	man	51895176

Homo sapiens	man	51895190
Homo sapiens	man	51895204
Homo sapiens	man	51895218
Homo sapiens	man	51895232
Homo sapiens	man	51895246
Homo sapiens	man	51895260
Homo sapiens	man	51895274
Homo sapiens	man	51895288
Homo sapiens	man	51895302
Homo sapiens	man	51895316
Homo sapiens	man	51895330
Homo sapiens	man	57903744
Homo sapiens	man	57903758
Homo sapiens	man	57903772
Homo sapiens	man	57903786
Homo sapiens	man	57903800
Homo sapiens	man	57903814
Homo sapiens	man	57903828
Homo sapiens	man	57903842
Homo sapiens	man	57903856
Homo sapiens	man	57903870
Homo sapiens	man	57903884
Homo sapiens	man	57903898
Homo sapiens	man	57903912
Homo sapiens	man	57903926
Homo sapiens	man	57903940
Homo sapiens	man	57903954
Homo sapiens	man	57903968
Homo sapiens	man	57903982
Homo sapiens	man	57903996
Homo sapiens	man	57904010
Homo sapiens	man	57904024
Homo sapiens	man	57904038
Homo sapiens	man	57904052
Homo sapiens	man	57904066
Homo sapiens	man	57904080
Homo sapiens	man	57904094
Homo sapiens	man	57904108
Homo sapiens	man	57904122
Homo sapiens	man	57904136
Homo sapiens	man	57904150
Homo sapiens	man	57904164
Homo sapiens	man	57904178
Homo sapiens	man	57904192
Homo sapiens	man	57904206
Homo sapiens	man	57904220
Homo sapiens	man	57904234
Homo sapiens	man	57904248
Homo sapiens	man	57904262
Homo sapiens	man	57904276
Homo sapiens	man	58615662
Homo sapiens	man	60257000
Homo sapiens	man	60257014
Homo sapiens	man	60257028
Homo sapiens	man	60257042
Homo sapiens	man	60257056
Homo sapiens	man	60257070
Homo sapiens	man	60257084
Homo sapiens	man	60257098

Homo sapiens	man	60257112
Homo sapiens	man	60257126
Homo sapiens	man	60257140
Homo sapiens	man	60257154
Homo sapiens	man	60257168
Homo sapiens	man	60257182
Homo sapiens	man	60257196
Homo sapiens	man	60257210
Homo sapiens	man	60257224
Homo sapiens	man	60257238
Homo sapiens	man	60257252
Homo sapiens	man	60257266
Homo sapiens	man	60257280
Homo sapiens	man	60257294
Homo sapiens	man	60257308
Homo sapiens	man	60257322
Homo sapiens	man	60257336
Homo sapiens	man	60257350
Homo sapiens	man	60257364
Homo sapiens	man	60257378
Homo sapiens	man	60257392
Homo sapiens	man	60257406
Homo sapiens	man	60257420
Homo sapiens	man	60257434
Homo sapiens	man	60257448
Homo sapiens	man	60257462
Homo sapiens	man	60257476
Homo sapiens	man	60257490
Homo sapiens	man	60257504
Homo sapiens	man	60257518
Homo sapiens	man	60257532
Homo sapiens	man	60257546
Homo sapiens	man	60257560
Homo sapiens	man	60257574
Homo sapiens	man	60257588
Homo sapiens	man	60257602
Homo sapiens	man	60257616
Homo sapiens	man	60257630
Homo sapiens	man	60257644
Homo sapiens	man	60257658
Homo sapiens	man	60257672
Homo sapiens	man	60257686
Homo sapiens	man	60257700
Homo sapiens	man	60257714
Homo sapiens	man	60257728
Homo sapiens	man	60257742
Homo sapiens	man	60257756
Homo sapiens	man	60257770
Homo sapiens	man	61287208
Homo sapiens	man	61287211
Homo sapiens	man	61287212
Homo sapiens	man	61287213
Homo sapiens	man	61287214
Homo sapiens	man	61287216
Homo sapiens	man	61287218
Homo sapiens	man	61287219
Homo sapiens	man	61287221
Homo sapiens	man	61287222
Homo sapiens	man	61287224

Homo sapiens	man	61287226
Homo sapiens	man	61287227
Homo sapiens	man	61287228
Homo sapiens	man	61287230
Homo sapiens	man	61287232
Homo sapiens	man	61287233
Homo sapiens	man	61287235
Homo sapiens	man	61287237
Homo sapiens	man	61287238
Homo sapiens	man	61287240
Homo sapiens	man	61287241
Homo sapiens	man	61287243
Homo sapiens	man	61287245
Homo sapiens	man	61287246
Homo sapiens	man	61287249
Homo sapiens	man	61287250
Homo sapiens	man	61287251
Homo sapiens	man	61287252
Homo sapiens	man	61287255
Homo sapiens	man	61287256
Homo sapiens	man	61287257
Homo sapiens	man	61287260
Homo sapiens	man	61287261
Homo sapiens	man	61287263
Homo sapiens	man	61287265
Homo sapiens	man	61287267
Homo sapiens	man	61287268
Homo sapiens	man	61287269
Homo sapiens	man	61287272
Homo sapiens	man	61287273
Homo sapiens	man	61287275
Homo sapiens	man	61287277
Homo sapiens	man	61287280
Homo sapiens	man	61287281
Homo sapiens	man	61287283
Homo sapiens	man	61287285
Homo sapiens	man	61287287
Homo sapiens	man	61287288
Homo sapiens	man	61287290
Homo sapiens	man	61287292
Homo sapiens	man	61287293
Homo sapiens	man	61287294
Homo sapiens	man	61287297
Homo sapiens	man	61287298
Homo sapiens	man	61287299
Homo sapiens	man	61287301
Homo sapiens	man	61287303
Homo sapiens	man	61287304
Homo sapiens	man	61287305
Homo sapiens	man	61287306
Homo sapiens	man	61287307
Homo sapiens	man	61287309
Homo sapiens	man	61287311
Homo sapiens	man	61287313
Homo sapiens	man	61287314
Homo sapiens	man	61287315
Homo sapiens	man	61287318
Homo sapiens	man	61287319
Homo sapiens	man	61287320

Homo sapiens	man	61287321
Homo sapiens	man	61287324
Homo sapiens	man	61287325
Homo sapiens	man	61287326
Homo sapiens	man	61287327
Homo sapiens	man	61287329
Homo sapiens	man	61287330
Homo sapiens	man	61287332
Homo sapiens	man	61287334
Homo sapiens	man	61287335
Homo sapiens	man	61287336
Homo sapiens	man	61287339
Homo sapiens	man	61287340
Homo sapiens	man	61287341
Homo sapiens	man	61287342
Homo sapiens	man	61287345
Homo sapiens	man	61287346
Homo sapiens	man	61287347
Homo sapiens	man	61287348
Homo sapiens	man	61287351
Homo sapiens	man	61287352
Homo sapiens	man	61287353
Homo sapiens	man	61287355
Homo sapiens	man	61287357
Homo sapiens	man	61287358
Homo sapiens	man	61287360
Homo sapiens	man	61287361
Homo sapiens	man	61287363
Homo sapiens	man	61287365
Homo sapiens	man	61287366
Homo sapiens	man	61287368
Homo sapiens	man	61287370
Homo sapiens	man	61287371
Homo sapiens	man	61287373
Homo sapiens	man	61287374
Homo sapiens	man	61287377
Homo sapiens	man	61287378
Homo sapiens	man	61287379
Homo sapiens	man	61287381
Homo sapiens	man	61287383
Homo sapiens	man	61287384
Homo sapiens	man	61287386
Homo sapiens	man	61287388
Homo sapiens	man	61287389
Homo sapiens	man	61287391
Homo sapiens	man	61287393
Homo sapiens	man	61287395
Homo sapiens	man	61287396
Homo sapiens	man	61287398
Homo sapiens	man	61287400
Homo sapiens	man	61287401
Homo sapiens	man	61287402
Homo sapiens	man	61287405
Homo sapiens	man	61287406
Homo sapiens	man	61287407
Homo sapiens	man	61287410
Homo sapiens	man	61287411
Homo sapiens	man	61287412
Homo sapiens	man	61287414

Homo sapiens	man	61287416
Homo sapiens	man	61287417
Homo sapiens	man	61287419
Homo sapiens	man	61287421
Homo sapiens	man	61287423
Homo sapiens	man	61287424
Homo sapiens	man	61287425
Homo sapiens	man	61287426
Homo sapiens	man	61287429
Homo sapiens	man	61287430
Homo sapiens	man	61287431
Homo sapiens	man	61287432
Homo sapiens	man	61287435
Homo sapiens	man	61287436
Homo sapiens	man	61287437
Homo sapiens	man	61287439
Homo sapiens	man	61287441
Homo sapiens	man	61287442
Homo sapiens	man	61287444
Homo sapiens	man	61287445
Homo sapiens	man	61287447
Homo sapiens	man	61287449
Homo sapiens	man	61287450
Homo sapiens	man	61287451
Homo sapiens	man	61287454
Homo sapiens	man	61287455
Homo sapiens	man	61287456
Homo sapiens	man	61287459
Homo sapiens	man	61287460
Homo sapiens	man	61287462
Homo sapiens	man	61287464
Homo sapiens	man	61287465
Homo sapiens	man	61287466
Homo sapiens	man	61287468
Homo sapiens	man	61287469
Homo sapiens	man	61287470
Homo sapiens	man	61287471
Homo sapiens	man	61287473
Homo sapiens	man	61287474
Homo sapiens	man	61287475
Homo sapiens	man	61287477
Homo sapiens	man	61287479
Homo sapiens	man	61287480
Homo sapiens	man	61287482
Homo sapiens	man	61287484
Homo sapiens	man	61287485
Homo sapiens	man	61287487
Homo sapiens	man	61287489
Homo sapiens	man	61287490
Homo sapiens	man	61287492
Homo sapiens	man	61287493
Homo sapiens	man	61287495
Homo sapiens	man	61287497
Homo sapiens	man	61287498
Homo sapiens	man	61287501
Homo sapiens	man	61287502
Homo sapiens	man	61287503
Homo sapiens	man	61287505
Homo sapiens	man	61287507

Homo sapiens	man	61287508
Homo sapiens	man	61287510
Homo sapiens	man	61287512
Homo sapiens	man	61287514
Homo sapiens	man	61287515
Homo sapiens	man	61287517
Homo sapiens	man	61287519
Homo sapiens	man	61287520
Homo sapiens	man	61287521
Homo sapiens	man	61287522
Homo sapiens	man	61287524
Homo sapiens	man	61287526
Homo sapiens	man	61287527
Homo sapiens	man	61287528
Homo sapiens	man	61287530
Homo sapiens	man	61287531
Homo sapiens	man	61287533
Homo sapiens	man	61287534
Homo sapiens	man	61287536
Homo sapiens	man	61287537
Homo sapiens	man	61287539
Homo sapiens	man	61287541
Homo sapiens	man	61287542
Homo sapiens	man	61287544
Homo sapiens	man	61287546
Homo sapiens	man	61287547
Homo sapiens	man	61287548
Homo sapiens	man	61287551
Homo sapiens	man	61287552
Homo sapiens	man	61287553
Homo sapiens	man	61287556
Homo sapiens	man	61287557
Homo sapiens	man	61287559
Homo sapiens	man	61287561
Homo sapiens	man	61287563
Homo sapiens	man	61287564
Homo sapiens	man	61287566
Homo sapiens	man	61287568
Homo sapiens	man	61287569
Homo sapiens	man	61287570
Homo sapiens	man	61287573
Homo sapiens	man	61287574
Homo sapiens	man	61287575
Homo sapiens	man	61287577
Homo sapiens	man	61287579
Homo sapiens	man	61287580
Homo sapiens	man	61287582
Homo sapiens	man	61287583
Homo sapiens	man	61287585
Homo sapiens	man	61287587
Homo sapiens	man	61287588
Homo sapiens	man	61287589
Homo sapiens	man	61287592
Homo sapiens	man	61287593
Homo sapiens	man	61287594
Homo sapiens	man	61287596
Homo sapiens	man	61287598
Homo sapiens	man	61287600
Homo sapiens	man	61287601

Homo sapiens	man	61287604
Homo sapiens	man	61287605
Homo sapiens	man	61287606
Homo sapiens	man	61287608
Homo sapiens	man	61287610
Homo sapiens	man	61287611
Homo sapiens	man	61287613
Homo sapiens	man	61287615
Homo sapiens	man	61287616
Homo sapiens	man	61287617
Homo sapiens	man	61287619
Homo sapiens	man	61287621
Homo sapiens	man	61287622
Homo sapiens	man	61287624
Homo sapiens	man	61287626
Homo sapiens	man	61287627
Homo sapiens	man	61287629
Homo sapiens	man	61287630
Homo sapiens	man	61287632
Homo sapiens	man	61287634
Homo sapiens	man	61287635
Homo sapiens	man	61287637
Homo sapiens	man	61287639
Homo sapiens	man	61287640
Homo sapiens	man	61287642
Homo sapiens	man	61287644
Homo sapiens	man	61287645
Homo sapiens	man	61287646
Homo sapiens	man	61287648
Homo sapiens	man	61287650
Homo sapiens	man	61287651
Homo sapiens	man	61287653
Homo sapiens	man	61287655
Homo sapiens	man	61287656
Homo sapiens	man	61287658
Homo sapiens	man	61287660
Homo sapiens	man	61287662
Homo sapiens	man	61287663
Homo sapiens	man	61287665
Homo sapiens	man	61287667
Homo sapiens	man	61287668
Homo sapiens	man	61287669
Homo sapiens	man	61287672
Homo sapiens	man	61287673
Homo sapiens	man	61287675
Homo sapiens	man	61287677
Homo sapiens	man	61287678
Homo sapiens	man	61287680
Homo sapiens	man	61287682
Homo sapiens	man	61287684
Homo sapiens	man	61287685
Homo sapiens	man	61287687
Homo sapiens	man	61287689
Homo sapiens	man	61287690
Homo sapiens	man	61287692
Homo sapiens	man	61287694
Homo sapiens	man	61287696
Homo sapiens	man	61287698
Homo sapiens	man	61287699

Homo sapiens	man	61287701
Homo sapiens	man	61287703
Homo sapiens	man	61287704
Homo sapiens	man	61287707
Homo sapiens	man	61287708
Homo sapiens	man	61287709
Homo sapiens	man	61287710
Homo sapiens	man	61287713
Homo sapiens	man	61287714
Homo sapiens	man	61287716
Homo sapiens	man	61287718
Homo sapiens	man	61287719
Homo sapiens	man	61287720
Homo sapiens	man	61287722
Homo sapiens	man	61287723
Homo sapiens	man	61287725
Homo sapiens	man	61287727
Homo sapiens	man	61287728
Homo sapiens	man	61287729
Homo sapiens	man	61287732
Homo sapiens	man	61287733
Homo sapiens	man	61287734
Homo sapiens	man	61287736
Homo sapiens	man	61287738
Homo sapiens	man	61287739
Homo sapiens	man	61287740
Homo sapiens	man	61287742
Homo sapiens	man	61287744
Homo sapiens	man	61287745
Homo sapiens	man	61287747
Homo sapiens	man	61287749
Homo sapiens	man	61287750
Homo sapiens	man	61287752
Homo sapiens	man	61287754
Homo sapiens	man	61287756
Homo sapiens	man	61287757
Homo sapiens	man	61287758
Homo sapiens	man	61287759
Homo sapiens	man	61287762
Homo sapiens	man	61287763
Homo sapiens	man	61287764
Homo sapiens	man	61287767
Homo sapiens	man	61287768
Homo sapiens	man	61287769
Homo sapiens	man	61287772
Homo sapiens	man	61287773
Homo sapiens	man	61287775
Homo sapiens	man	61287777
Homo sapiens	man	61287778
Homo sapiens	man	61287780
Homo sapiens	man	61287782
Homo sapiens	man	61287784
Homo sapiens	man	61287785
Homo sapiens	man	61287788
Homo sapiens	man	61287789
Homo sapiens	man	61287790
Homo sapiens	man	61287793
Homo sapiens	man	61287794
Homo sapiens	man	61287795

Homo sapiens	man	61287798
Homo sapiens	man	61287799
Homo sapiens	man	61287800
Homo sapiens	man	61287802
Homo sapiens	man	61287804
Homo sapiens	man	61287806
Homo sapiens	man	61287808
Homo sapiens	man	61287809
Homo sapiens	man	61287811
Homo sapiens	man	61287813
Homo sapiens	man	61287814
Homo sapiens	man	61287816
Homo sapiens	man	61287818
Homo sapiens	man	61287819
Homo sapiens	man	61287821
Homo sapiens	man	61287823
Homo sapiens	man	61287825
Homo sapiens	man	61287826
Homo sapiens	man	61287829
Homo sapiens	man	61287830
Homo sapiens	man	61287831
Homo sapiens	man	61287834
Homo sapiens	man	61287835
Homo sapiens	man	61287836
Homo sapiens	man	61287838
Homo sapiens	man	61287840
Homo sapiens	man	61287841
Homo sapiens	man	61287843
Homo sapiens	man	61287845
Homo sapiens	man	61287847
Homo sapiens	man	61287849
Homo sapiens	man	61287850
Homo sapiens	man	61287851
Homo sapiens	man	61287854
Homo sapiens	man	61287855
Homo sapiens	man	61287856
Homo sapiens	man	61287858
Homo sapiens	man	61287860
Homo sapiens	man	61287861
Homo sapiens	man	61287862
Homo sapiens	man	61287865
Homo sapiens	man	61287866
Homo sapiens	man	61287867
Homo sapiens	man	61287869
Homo sapiens	man	61287871
Homo sapiens	man	61287872
Homo sapiens	man	61287873
Homo sapiens	man	61287876
Homo sapiens	man	61287877
Homo sapiens	man	61287879
Homo sapiens	man	61287881
Homo sapiens	man	61287882
Homo sapiens	man	61287884
Homo sapiens	man	61287886
Homo sapiens	man	61287887
Homo sapiens	man	61287889
Homo sapiens	man	61287892
Homo sapiens	man	61287893
Homo sapiens	man	61287894

Homo sapiens	man	61287897
Homo sapiens	man	61287898
Homo sapiens	man	61287899
Homo sapiens	man	61287902
Homo sapiens	man	61287903
Homo sapiens	man	61287904
Homo sapiens	man	61287906
Homo sapiens	man	61287908
Homo sapiens	man	61287909
Homo sapiens	man	61287912
Homo sapiens	man	61287913
Homo sapiens	man	61287914
Homo sapiens	man	61287916
Homo sapiens	man	61287918
Homo sapiens	man	61287919
Homo sapiens	man	61287921
Homo sapiens	man	61287923
Homo sapiens	man	61287924
Homo sapiens	man	61287925
Homo sapiens	man	61287927
Homo sapiens	man	61287929
Homo sapiens	man	61287930
Homo sapiens	man	61287932
Homo sapiens	man	61287955
Homo sapiens	man	61287957
Homo sapiens	man	61287958
Homo sapiens	man	61287960
Homo sapiens	man	61287961
Homo sapiens	man	61287963
Homo sapiens	man	61287964
Homo sapiens	man	61287966
Homo sapiens	man	61287968
Homo sapiens	man	61287969
Homo sapiens	man	61287970
Homo sapiens	man	61287971
Homo sapiens	man	61287974
Homo sapiens	man	61287975
Homo sapiens	man	61287976
Homo sapiens	man	61287977
Homo sapiens	man	61287980
Homo sapiens	man	61287981
Homo sapiens	man	61287982
Homo sapiens	man	61287983
Homo sapiens	man	61287985
Homo sapiens	man	61287987
Homo sapiens	man	61287988
Homo sapiens	man	61287990
Homo sapiens	man	61287992
Homo sapiens	man	61287994
Homo sapiens	man	61287995
Homo sapiens	man	61287997
Homo sapiens	man	61287999
Homo sapiens	man	61288000
Homo sapiens	man	61288001
Homo sapiens	man	61288002
Homo sapiens	man	61288004
Homo sapiens	man	61288006
Homo sapiens	man	61288007
Homo sapiens	man	61288008

Homo sapiens	man	61288010
Homo sapiens	man	61288012
Homo sapiens	man	61288013
Homo sapiens	man	61288014
Homo sapiens	man	61288015
Homo sapiens	man	61288017
Homo sapiens	man	61288019
Homo sapiens	man	61288020
Homo sapiens	man	61288021
Homo sapiens	man	61288022
Homo sapiens	man	61288023
Homo sapiens	man	61288024
Homo sapiens	man	61288027
Homo sapiens	man	61288028
Homo sapiens	man	61288029
Homo sapiens	man	61288032
Homo sapiens	man	61288033
Homo sapiens	man	61288035
Homo sapiens	man	61288037
Homo sapiens	man	61288038
Homo sapiens	man	61288039
Homo sapiens	man	61288040
Homo sapiens	man	61288042
Homo sapiens	man	61288044
Homo sapiens	man	61288045
Homo sapiens	man	61288046
Homo sapiens	man	61288047
Homo sapiens	man	61288050
Homo sapiens	man	61288051
Homo sapiens	man	61288053
Homo sapiens	man	61288054
Homo sapiens	man	61288056
Homo sapiens	man	61288057
Homo sapiens	man	61288059
Homo sapiens	man	61288061
Homo sapiens	man	61288063
Homo sapiens	man	61288064
Homo sapiens	man	61288066
Homo sapiens	man	61288068
Homo sapiens	man	61288069
Homo sapiens	man	61288070
Homo sapiens	man	61288073
Homo sapiens	man	61288074
Homo sapiens	man	61288075
Homo sapiens	man	61288078
Homo sapiens	man	61288079
Homo sapiens	man	61288080
Homo sapiens	man	61288081
Homo sapiens	man	61288082
Homo sapiens	man	61288083
Homo sapiens	man	61288086
Homo sapiens	man	61288087
Homo sapiens	man	61288088
Homo sapiens	man	61288089
Homo sapiens	man	61288092
Homo sapiens	man	61288093
Homo sapiens	man	61288095
Homo sapiens	man	61288096
Homo sapiens	man	61288097

Homo sapiens	man	61288099
Homo sapiens	man	61288101
Homo sapiens	man	61288102
Homo sapiens	man	61288103
Homo sapiens	man	61288105
Homo sapiens	man	61288107
Homo sapiens	man	61288108
Homo sapiens	man	61288109
Homo sapiens	man	61288111
Homo sapiens	man	61288113
Homo sapiens	man	61288114
Homo sapiens	man	61288115
Homo sapiens	man	61288117
Homo sapiens	man	61288119
Homo sapiens	man	61288120
Homo sapiens	man	61288121
Homo sapiens	man	61288123
Homo sapiens	man	61288125
Homo sapiens	man	61288126
Homo sapiens	man	61288128
Homo sapiens	man	61288130
Homo sapiens	man	61288132
Homo sapiens	man	61288133
Homo sapiens	man	61288135
Homo sapiens	man	61288137
Homo sapiens	man	61288138
Homo sapiens	man	61288141
Homo sapiens	man	61288142
Homo sapiens	man	61288144
Homo sapiens	man	61288146
Homo sapiens	man	61288147
Homo sapiens	man	61288148
Homo sapiens	man	61288151
Homo sapiens	man	61288152
Homo sapiens	man	61288153
Homo sapiens	man	61288155
Homo sapiens	man	61288157
Homo sapiens	man	61288158
Homo sapiens	man	61288160
Homo sapiens	man	61288161
Homo sapiens	man	61288163
Homo sapiens	man	61288165
Homo sapiens	man	61288166
Homo sapiens	man	61288167
Homo sapiens	man	61288170
Homo sapiens	man	61288171
Homo sapiens	man	61288172
Homo sapiens	man	61288175
Homo sapiens	man	61288176
Homo sapiens	man	61288178
Homo sapiens	man	61288180
Homo sapiens	man	61288181
Homo sapiens	man	61288183
Homo sapiens	man	61288185
Homo sapiens	man	61288186
Homo sapiens	man	61288188
Homo sapiens	man	61288190
Homo sapiens	man	61288191
Homo sapiens	man	61288193

Homo sapiens	man	61288195
Homo sapiens	man	61288196
Homo sapiens	man	61288198
Homo sapiens	man	61288200
Homo sapiens	man	61288202
Homo sapiens	man	61288203
Homo sapiens	man	61288205
Homo sapiens	man	61288206
Homo sapiens	man	61288208
Homo sapiens	man	61288210
Homo sapiens	man	61288211
Homo sapiens	man	61288213
Homo sapiens	man	61288215
Homo sapiens	man	61288216
Homo sapiens	man	61288218
Homo sapiens	man	61288220
Homo sapiens	man	61288222
Homo sapiens	man	61288223
Homo sapiens	man	61288225
Homo sapiens	man	61288227
Homo sapiens	man	61288228
Homo sapiens	man	61288229
Homo sapiens	man	61288232
Homo sapiens	man	61288233
Homo sapiens	man	61288236
Homo sapiens	man	61288237
Homo sapiens	man	61288239
Homo sapiens	man	61288241
Homo sapiens	man	61288242
Homo sapiens	man	61288245
Homo sapiens	man	61288246
Homo sapiens	man	61288248
Homo sapiens	man	61288250
Homo sapiens	man	61288252
Homo sapiens	man	61288253
Homo sapiens	man	61288254
Homo sapiens	man	61288257
Homo sapiens	man	61288258
Homo sapiens	man	61288259
Homo sapiens	man	61288262
Homo sapiens	man	61288263
Homo sapiens	man	61288265
Homo sapiens	man	61288267
Homo sapiens	man	61288269
Homo sapiens	man	61288271
Homo sapiens	man	61288272
Homo sapiens	man	61288274
Homo sapiens	man	61288276
Homo sapiens	man	61288277
Homo sapiens	man	61288279
Homo sapiens	man	61288281
Homo sapiens	man	61288282
Homo sapiens	man	61288284
Homo sapiens	man	61288286
Homo sapiens	man	61288287
Homo sapiens	man	61288290
Homo sapiens	man	61288291
Homo sapiens	man	61288293
Homo sapiens	man	61288295

Homo sapiens	man	61288296
Homo sapiens	man	61288297
Homo sapiens	man	61288300
Homo sapiens	man	61288301
Homo sapiens	man	61288303
Homo sapiens	man	61288305
Homo sapiens	man	61288306
Homo sapiens	man	61288307
Homo sapiens	man	61288310
Homo sapiens	man	61288311
Homo sapiens	man	61288313
Homo sapiens	man	61288315
Homo sapiens	man	61393479
Homo sapiens	man	61393497
Homo sapiens	man	61393513
Homo sapiens	man	61724355
Homo sapiens	man	61724582
Homo sapiens	man	61743586
Homo sapiens	man	66356283
Homo sapiens	man	66356284
Homo sapiens	man	66356285
Homo sapiens	man	66356286
Homo sapiens	man	66394216
Homo sapiens	man	69938881
Homo sapiens	man	69938882
Homo sapiens	man	69938883
Homo sapiens	man	69938884
Homo sapiens	man	69938885
Homo sapiens	man	69938886
Homo sapiens	man	69938887
Homo sapiens	man	69938888
Homo sapiens	man	70955706
Homo sapiens	man	70955708
Homo sapiens	man	70955710
Homo sapiens	man	70955712
Homo sapiens	man	70955714
Homo sapiens	man	70955716
Homo sapiens	man	70955718
Homo sapiens	man	70955720
Homo sapiens	man	70955722
Homo sapiens	man	70955724
Homo sapiens	man	70955726
Homo sapiens	man	70955728
Homo sapiens	man	70955730
Homo sapiens	man	70955732
Homo sapiens	man	70955734
Homo sapiens	man	70955736
Homo sapiens	man	70955738
Homo sapiens	man	70955740
Homo sapiens	man	70955742
Homo sapiens	man	70955744
Homo sapiens	man	70955746
Homo sapiens	man	70955748
Homo sapiens	man	70955750
Homo sapiens	man	70955752
Homo sapiens	man	70955754
Homo sapiens	man	70955756
Homo sapiens	man	70955758
Homo sapiens	man	70955760

Homo sapiens	man	70955762
Homo sapiens	man	70955764
Homo sapiens	man	70955766
Homo sapiens	man	70955768
Homo sapiens	man	70955770
Homo sapiens	man	70955772
Homo sapiens	man	70955774
Homo sapiens	man	70955776
Homo sapiens	man	70955778
Homo sapiens	man	70955780
Homo sapiens	man	70955782
Homo sapiens	man	70955784
Homo sapiens	man	70955786
Homo sapiens	man	70955788
Homo sapiens	man	70955790
Homo sapiens	man	70955792
Homo sapiens	man	70955794
Homo sapiens	man	70955796
Homo sapiens	man	70955798
Homo sapiens	man	70955800
Homo sapiens	man	70955802
Homo sapiens	man	70955804
Homo sapiens	man	70955806
Homo sapiens	man	70955808
Homo sapiens	man	70955810
Homo sapiens	man	70955812
Homo sapiens	man	70955814
Homo sapiens	man	70955816
Homo sapiens	man	70955818
Homo sapiens	man	70955820
Homo sapiens	man	70955822
Homo sapiens	man	70955824
Homo sapiens	man	70955826
Homo sapiens	man	70955828
Homo sapiens	man	70955830
Homo sapiens	man	70955832
Homo sapiens	man	70955834
Homo sapiens	man	70955836
Homo sapiens	man	70955838
Homo sapiens	man	70955840
Homo sapiens	man	70955842
Homo sapiens	man	70955844
Homo sapiens	man	70955846
Homo sapiens	man	70955848
Homo sapiens	man	70955850
Homo sapiens	man	70955852
Homo sapiens	man	70955854
Homo sapiens	man	70955856
Homo sapiens	man	70955858
Homo sapiens	man	70955860
Homo sapiens	man	70955862
Homo sapiens	man	70955864
Homo sapiens	man	70955866
Homo sapiens	man	70955868
Homo sapiens	man	70955870
Homo sapiens	man	70955872
Homo sapiens	man	70955874
Homo sapiens	man	70955876
Homo sapiens	man	70955878

Homo sapiens	man	70955880
Homo sapiens	man	70955882
Homo sapiens	man	70955884
Homo sapiens	man	70955886
Homo sapiens	man	70955888
Homo sapiens	man	70955890
Homo sapiens	man	70955892
Homo sapiens	man	70955894
Homo sapiens	man	70955896
Homo sapiens	man	70955898
Homo sapiens	man	70955900
Homo sapiens	man	70955902
Homo sapiens	man	70955904
Homo sapiens	man	70955906
Homo sapiens	man	70955908
Homo sapiens	man	70955910
Homo sapiens	man	70955912
Homo sapiens	man	70955914
Homo sapiens	man	70955916
Homo sapiens	man	70955918
Homo sapiens	man	70955920
Homo sapiens	man	70955922
Homo sapiens	man	70955924
Homo sapiens	man	70955926
Homo sapiens	man	70955928
Homo sapiens	man	70955930
Homo sapiens	man	70955932
Homo sapiens	man	70955934
Homo sapiens	man	70955936
Homo sapiens	man	70955938
Homo sapiens	man	70955940
Homo sapiens	man	70955942
Homo sapiens	man	70955944
Homo sapiens	man	70955946
Homo sapiens	man	70955948
Homo sapiens	man	70955950
Homo sapiens	man	70955952
Homo sapiens	man	70955954
Homo sapiens	man	70955956
Homo sapiens	man	70955958
Homo sapiens	man	70955960
Homo sapiens	man	70955962
Homo sapiens	man	70955964
Homo sapiens	man	70955966
Homo sapiens	man	70955968
Homo sapiens	man	70955970
Homo sapiens	man	70955972
Homo sapiens	man	70955974
Homo sapiens	man	70955976
Homo sapiens	man	70955978
Homo sapiens	man	70955980
Homo sapiens	man	70955982
Homo sapiens	man	70955984
Homo sapiens	man	70955986
Homo sapiens	man	70955988
Homo sapiens	man	70955990
Homo sapiens	man	70955992
Homo sapiens	man	70955994
Homo sapiens	man	70955996

Homo sapiens	man	70955998
Homo sapiens	man	70956000
Homo sapiens	man	70956002
Homo sapiens	man	70956004
Homo sapiens	man	70956006
Homo sapiens	man	70956008
Homo sapiens	man	70956010
Homo sapiens	man	70956012
Homo sapiens	man	70956014
Homo sapiens	man	70956016
Homo sapiens	man	70956018
Homo sapiens	man	70956020
Homo sapiens	man	70956022
Homo sapiens	man	70956024
Homo sapiens	man	70956026
Homo sapiens	man	70956028
Homo sapiens	man	70956030
Homo sapiens	man	70956032
Homo sapiens	man	70956034
Homo sapiens	man	70956036
Homo sapiens	man	70956038
Homo sapiens	man	70956040
Homo sapiens	man	70956042
Homo sapiens	man	70956044
Homo sapiens	man	70956046
Homo sapiens	man	70956048
Homo sapiens	man	70956050
Homo sapiens	man	70956052
Homo sapiens	man	70956054
Homo sapiens	man	70956056
Homo sapiens	man	70956058
Homo sapiens	man	70956060
Homo sapiens	man	70956062
Homo sapiens	man	70956064
Homo sapiens	man	70956066
Homo sapiens	man	70956068
Homo sapiens	man	70956070
Homo sapiens	man	70956072
Homo sapiens	man	70956074
Homo sapiens	man	70956076
Homo sapiens	man	70956078
Homo sapiens	man	70956080
Homo sapiens	man	70956082
Homo sapiens	man	70956084
Homo sapiens	man	70956086
Homo sapiens	man	70956088
Homo sapiens	man	70956090
Homo sapiens	man	70956092
Homo sapiens	man	70956094
Homo sapiens	man	70956096
Homo sapiens	man	70956098
Homo sapiens	man	70956100
Homo sapiens	man	70956102
Homo sapiens	man	70956104
Homo sapiens	man	70956106
Homo sapiens	man	70956108
Homo sapiens	man	70956110
Homo sapiens	man	70956112
Homo sapiens	man	70956114

Homo sapiens	man	70956116
Homo sapiens	man	70956118
Homo sapiens	man	70956120
Homo sapiens	man	70956122
Homo sapiens	man	70956124
Homo sapiens	man	70956126
Homo sapiens	man	70956128
Homo sapiens	man	70956130
Homo sapiens	man	70956132
Homo sapiens	man	70956134
Homo sapiens	man	70956136
Homo sapiens	man	70956138
Homo sapiens	man	70956140
Homo sapiens	man	70956142
Homo sapiens	man	70956144
Homo sapiens	man	70956146
Homo sapiens	man	70956148
Homo sapiens	man	70956150
Homo sapiens	man	70956152
Homo sapiens	man	70956154
Homo sapiens	man	70956156
Homo sapiens	man	70956158
Homo sapiens	man	70956160
Homo sapiens	man	70956162
Homo sapiens	man	70956164
Homo sapiens	man	70956166
Homo sapiens	man	70956168
Homo sapiens	man	70956170
Homo sapiens	man	70956172
Homo sapiens	man	70956174
Homo sapiens	man	70956176
Homo sapiens	man	70956178
Homo sapiens	man	70956180
Homo sapiens	man	70956182
Homo sapiens	man	70956184
Homo sapiens	man	70956186
Homo sapiens	man	70956188
Homo sapiens	man	70956190
Homo sapiens	man	70956192
Homo sapiens	man	70956194
Homo sapiens	man	70956196
Homo sapiens	man	70956198
Homo sapiens	man	70956200
Homo sapiens	man	70956202
Homo sapiens	man	70956204
Homo sapiens	man	70956206
Homo sapiens	man	70956208
Homo sapiens	man	70956210
Homo sapiens	man	70956212
Homo sapiens	man	70956214
Homo sapiens	man	70956216
Homo sapiens	man	70956218
Homo sapiens	man	70956220
Homo sapiens	man	70956222
Homo sapiens	man	70956224
Homo sapiens	man	70956226
Homo sapiens	man	70956228
Homo sapiens	man	70956230
Homo sapiens	man	70956232

Homo sapiens	man	70956234
Homo sapiens	man	70956236
Homo sapiens	man	70956238
Homo sapiens	man	70956240
Homo sapiens	man	70956242
Homo sapiens	man	70956244
Homo sapiens	man	70956246
Homo sapiens	man	70956248
Homo sapiens	man	70956250
Homo sapiens	man	70956252
Homo sapiens	man	70956254
Homo sapiens	man	70956256
Homo sapiens	man	70956258
Homo sapiens	man	71373130
Homo sapiens	man	71373144
Homo sapiens	man	71373158
Homo sapiens	man	71373172
Homo sapiens	man	71373186
Homo sapiens	man	71373200
Homo sapiens	man	71373214
Homo sapiens	man	71373228
Homo sapiens	man	71373242
Homo sapiens	man	71373256
Homo sapiens	man	71373270
Homo sapiens	man	71373284
Homo sapiens	man	71373298
Homo sapiens	man	71373312
Homo sapiens	man	71979976
Homo sapiens	man	71979990
Homo sapiens	man	71980004
Homo sapiens	man	71980018
Homo sapiens	man	71980032
Homo sapiens	man	71980046
Homo sapiens	man	71980060
Homo sapiens	man	74475784
Homo sapiens	man	74475798
Homo sapiens	man	74475812
Homo sapiens	man	74475826
Homo sapiens	man	74475840
Homo sapiens	man	75905871
Homo sapiens	man	75905872
Homo sapiens	man	75905873
Homo sapiens	man	75905874
Homo sapiens	man	75905875
Homo sapiens	man	75905876
Homo sapiens	man	75905877
Homo sapiens	man	75905878
Homo sapiens	man	75905879
Homo sapiens	man	75905880
Homo sapiens	man	75905881
Homo sapiens	man	75905882
Homo sapiens	man	75905883
Homo sapiens	man	78498963
Homo sapiens	man	78498977
Homo sapiens	man	78498991
Homo sapiens	man	78499005
Homo sapiens	man	78499019
Homo sapiens	man	78499033
Homo sapiens	man	78499047

Homo sapiens	man	78499061
Homo sapiens	man	78499075
Homo sapiens	man	78499089
Homo sapiens	man	78499103
Homo sapiens	man	78499117
Homo sapiens	man	78499131
Homo sapiens	man	78499145
Homo sapiens	man	78499159
Homo sapiens	man	78499173
Homo sapiens	man	78499187
Homo sapiens	man	78499201
Homo sapiens	man	78499215
Homo sapiens	man	78499229
Homo sapiens	man	78499243
Homo sapiens	man	78499257
Homo sapiens	man	78499271
Homo sapiens	man	78775891
Homo sapiens	man	78775905
Homo sapiens	man	78775919
Homo sapiens	man	78775933
Homo sapiens	man	78775947
Homo sapiens	man	78775961
Homo sapiens	man	78775975
Homo sapiens	man	78775989
Homo sapiens	man	78776003
Homo sapiens	man	78776017
Homo sapiens	man	78776031
Homo sapiens	man	78776045
Homo sapiens	man	78776059
Homo sapiens	man	78776073
Homo sapiens	man	78776087
Homo sapiens	man	78776101
Homo sapiens	man	78776115
Homo sapiens	man	78776129
Homo sapiens	man	78776143
Homo sapiens	man	78776157
Homo sapiens	man	82792095
Homo sapiens	man	82792234
Homo sapiens	man	82792248
Homo sapiens	man	82792262
Homo sapiens	man	82792276
Homo sapiens	man	82792290
Homo sapiens	man	82792304
Homo sapiens	man	82792318
Homo sapiens	man	82792332
Homo sapiens	man	82792346
Homo sapiens	man	82792360
Homo sapiens	man	82792374
Homo sapiens	man	82792388
Homo sapiens	man	82792402
Homo sapiens	man	82792416
Homo sapiens	man	82792430
Homo sapiens	man	82792444
Homo sapiens	man	82792458
Homo sapiens	man	82792472
Homo sapiens	man	82792486
Homo sapiens	man	82792500
Homo sapiens	man	82792514
Homo sapiens	man	82792528

Homo sapiens	man	82792542
Homo sapiens	man	82792556
Homo sapiens	man	82792570
Homo sapiens	man	82792584
Homo sapiens	man	82792598
Homo sapiens	man	82792612
Homo sapiens	man	82792626
Homo sapiens	man	82792640
Homo sapiens	man	84682334
Homo sapiens	man	84682348
Homo sapiens	man	84682362
Homo sapiens	man	84682376
Homo sapiens	man	84682390
Homo sapiens	man	84682418
Homo sapiens	man	84682432
Homo sapiens	man	84682446
Homo sapiens	man	84682460
Homo sapiens	man	84682474
Homo sapiens	man	84682488
Homo sapiens	man	84682502
Homo sapiens	man	84682516
Homo sapiens	man	84682530
Homo sapiens	man	84682544
Homo sapiens	man	84682558
Homo sapiens	man	84682572
Homo sapiens	man	84682586
Homo sapiens	man	84682600
Homo sapiens	man	84682614
Homo sapiens	man	84682628
Homo sapiens	man	84682642
Homo sapiens	man	84682656
Homo sapiens	man	84682670
Homo sapiens	man	84682684
Homo sapiens	man	84682698
Homo sapiens	man	84682712
Homo sapiens	man	84682726
Homo sapiens	man	84682740
Homo sapiens	man	84682754
Homo sapiens	man	84682768
Homo sapiens	man	84682782
Homo sapiens	man	85541074
Homo sapiens	man	86450429
Homo sapiens	man	86450443
Homo sapiens	man	86450457
Homo sapiens	man	86450471
Homo sapiens	man	86450485
Homo sapiens	man	86450499
Homo sapiens	man	86450513
Homo sapiens	man	86450527
Homo sapiens	man	86450541
Homo sapiens	man	86450555
Homo sapiens	man	86450569
Homo sapiens	man	86450583
Homo sapiens	man	86450597
Homo sapiens	man	86450611
Homo sapiens	man	86450625
Homo sapiens	man	86450639
Homo sapiens	man	86450653
Homo sapiens	man	86450667

Homo sapiens	man	86450681
Homo sapiens	man	86450695
Homo sapiens	man	88942033
Homo sapiens	man	89214181
Homo sapiens	man	89214182
Homo sapiens	man	89214183
Homo sapiens	man	89214184
Homo sapiens	man	89214185
Homo sapiens	man	89473620
Homo sapiens	man	90577103
Homo sapiens	man	90819897
Homo sapiens	man	90819911
Homo sapiens	man	90819925
Homo sapiens	man	93117374
Homo sapiens	man	93280536
Homo sapiens	man	93280537
Homo sapiens	man	93280538
Homo sapiens	man	93280539
Homo sapiens	man	93280540
Homo sapiens	man	93280541
Homo sapiens	man	93280542
Homo sapiens	man	93280543
Homo sapiens	man	94315496
Homo sapiens	man	94315510
Homo sapiens	man	94449820
Homo sapiens	man	94449823
Homo sapiens	man	94449824
Homo sapiens	man	94449825
Homo sapiens	man	94449827
Homo sapiens	man	94449830
Homo sapiens	man	94449834
Homo sapiens	man	94449835
Homo sapiens	man	94449836
Homo sapiens	man	94449839
Homo sapiens	man	99079826
Hylobates lar	white-handed gibbon	5835820
Hyperoodon ampullatus	northern bottlenose whale	38707612
Inia geoffrensis	pink river dolphin	38707450
Isoodon macrourus	northern brown bandicoot	13676803
Isoodon macrourus	northern brown bandicoot	13786588
Jaculus jaculus	lesser Egyptian jerboa	40805123
Kogia breviceps	pygmy sperm whale	38707548
Lagenorhynchus albirostris	white-beaked dolphin	38707534
Lagorchestes hirsutus	rufous hare-wallaby	108793336
Lagorchestes hirsutus	rufous hare-wallaby	94481201
Lama pacos	alpaca	9755345
Lemur catta	ring-tailed lemur	21449875
Lepus europaeus	European hare	21492444
Lipotes vexillifer	Yangtze River dolphin	55274416
Lipotes vexillifer	Yangtze River dolphin	83309071
Loxodonta africana	African savannah elephant	6137801
Loxodonta africana	African savannah elephant	86278445
Macaca mulatta	rhesus monkeys	47156210
Macaca mulatta	rhesus monkeys	49146236
Macaca sylvanus	Barbary macaque	14010693
Macropus robustus	wallaroo	5835359
Macroscelides proboscideus	short-eared elephant shrew	21449903
Macrotis lagotis	bilby	56548761
Mammuthus primigenius	woolly mammoth	75914850

Mammuthus primigenius	woolly mammoth	81230401
Mammuthus primigenius	woolly mammoth	86278444
Manis tetradactyla	long-tailed pangolin	21449973
Megaptera novaeangliae	humpback whale	62086580
Megaptera novaeangliae	humpback whale	62184326
Metachirus nudicaudatus	brown four-eyed opossum	56548733
Microtus kikuchii	Taiwan vole	14599791
Microtus kikuchii	Taiwan vole	15079217
Microtus rossiaemeridionalis	southern vole	107736027
Microtus rossiaemeridionalis	southern vole	63081251
Mogera wogura	Japanese mole	32469165
Mogera wogura	Japanese mole	32526814
Monodelphis domestica	gray short-tailed opossum	52547305
Monodon monoceros	narwhal	38707570
Muntiacus crinifrons	black muntjac	28569617
Muntiacus crinifrons	black muntjac	28867217
Muntiacus muntjak	muntjak	28207776
Muntiacus muntjak	muntjak	28301754
Muntiacus reevesi	Reeves's muntjac	21842389
Muntiacus reevesi	Reeves's muntjac	21908045
Mus musculus	mouse	10336604
Mus musculus	mouse	33112617
Mus musculus	mouse	33115104
Mus musculus	mouse	342520
Mus musculus	mouse	34538597
Mus musculus	mouse	38488898
Mus musculus	mouse	41386985
Mus musculus	mouse	41386999
Mus musculus	mouse	41387013
Mus musculus	mouse	41387027
Mus musculus	mouse	62461709
Mus musculus	mouse	68349586
Mus musculus	mouse	68349600
Mus musculus	mouse	7798599
Mus musculus	mouse	7804453
Mus musculus	mouse	8096291
Mus musculus domesticus	western European house mouse	62198713
Mus musculus domesticus	western European house mouse	7770098
Mus musculus molossinus	Japanese wild mouse	50302071
Mus musculus molossinus	Japanese wild mouse	62184368
Myoxus glis	fat dormouse	5835484
Mystacina tuberculata	New Zealand lesser short-tailed bat	61660104
Mystacina tuberculata	New Zealand lesser short-tailed bat	62184382
Nannospalax ehrenbergi	Palestine mole rat	40805109
Nasalis larvatus	proboscis monkey	109689539
Nasalis larvatus	proboscis monkey	88174246
Notoryctes typhlops	marsupial mole	56548691
Nycticebus coucang	slow loris	14010665
Ochotona collaris	N/A	14599763
Ochotona collaris	N/A	15055558
Ochotona princeps	southern American pika	41353095
Odobenus rosmarus rosmarus	Atlantic walrus	34577125
Ornithorhynchus anatinus	platypus	1469249
Ornithorhynchus anatinus	platypus	5836058
Orycteropus afer	aardvark	5835764
Oryctolagus cuniculus	rabbits	5835526
Ovis aries	wild sheep	56713965
Ovis aries	wild sheep	5835554
Pan paniscus	pygmy chimpanzee	5835135

Pan troglodytes	chimpanzee	1262390
Pan troglodytes	chimpanzee	5835121
Pantholops hodgsonii	chiru	74419055
Pantholops hodgsonii	chiru	77020040
Papio hamadryas	western baboon	4049475
Papio hamadryas	western baboon	5835638
Perameles gunnii	eastern barred bandicoot	56548705
Petaurus breviceps	sugar glider	108793266
Petaurus breviceps	sugar glider	94481187
Phalanger interpositus	Stein's cuscus	108793518
Phalanger interpositus	Stein's cuscus	94481215
Phascogale tapoatafa	brush-tailed phascogale	56548775
Phascolarctos cinereus	koala	108793308
Phascolarctos cinereus	koala	94481159
Phoca vitulina	harbor seal	13431
Phoca vitulina	harbor seal	5834857
Phocoena phocoena	harbor porpoise	38707464
Physeter catodon	sperm whale	34582601
Pipistrellus abramus	Japanese house bat	16416436
Pipistrellus abramus	Japanese house bat	42632257
Platanista minor	Indus River dolphin	38707626
Pongo pygmaeus	orangutan	5835163
Pongo pygmaeus abelii	Sumatran orangutan	5835834
Pontoporia blainvillei	franciscana	38707584
Potorous tridactylus	potoroo	56548663
Presbytis melalophos	mitred leaf monkey	109689581
Presbytis melalophos	mitred leaf monkey	88174260
Procavia capensis	rock hyrax	32187980
Procavia capensis	rock hyrax	32402525
Procolobus badius	red colobus	109689609
Procolobus badius	red colobus	88174288
Pseudocheirus peregrinus	common ring-tailed possum	56548677
Pteropus dasymallus	Ryukyu flying fox	11231066
Pteropus dasymallus	Ryukyu flying fox	11386118
Pteropus scapulatus	little red flying fox	11545675
Pteropus scapulatus	little red flying fox	11602891
Pygathrix nemaeus	dove langur	109689553
Pygathrix nemaeus	dove langur	88174302
Pygathrix roxellana	golden snub-nosed monkey	109689567
Pygathrix roxellana	golden snub-nosed monkey	88174274
Rangifer tarandus	reindeer	84095017
Rangifer tarandus	reindeer	84488594
Rattus norvegicus	rats	110189662
Rattus norvegicus	rats	110189714
Rattus norvegicus	rats	26983975
Rattus norvegicus	rats	54145371
Rhinoceros unicornis	greater Indian rhinoceros	5835331
Rhinolophus monoceros	N/A	15420931
Rhinolophus monoceros	N/A	42717961
Rhinolophus pumilus	Okinawa least horseshoe bat	16416408
Rhinolophus pumilus	Okinawa least horseshoe bat	42632271
Rhyncholestes raphanurus	Chilean shrew opossum	45685677
Rousettus aegyptiacus	Egyptian rousette	73807999
Rousettus aegyptiacus	Egyptian rousette	74310519
Sciurus vulgaris	Eurasian red squirrel	8573078
Semnopithecus entellus	Hanuman langur	109689595
Semnopithecus entellus	Hanuman langur	88174232
Sminthopsis crassicaudata	fat-tailed dunnart	55416202
Sminthopsis crassicaudata	fat-tailed dunnart	83309029

Sminthopsis douglasi	Julia Creek dunnart	56548747
Sorex unguiculatus	long-clawed shrew	16416422
Sorex unguiculatus	long-clawed shrew	42632229
Sus scrofa	wild boar	33320683
Sus scrofa	wild boar	33320697
Sus scrofa	wild boar	33320711
Sus scrofa	wild boar	33320725
Sus scrofa	wild boar	33320739
Sus scrofa	wild boar	33320753
Sus scrofa	wild boar	33320767
Sus scrofa	wild boar	33320781
Sus scrofa	wild boar	33320795
Sus scrofa	wild boar	33320809
Sus scrofa	wild boar	33320823
Sus scrofa	wild boar	33320837
Sus scrofa	wild boar	33320851
Sus scrofa	wild boar	33320865
Sus scrofa	wild boar	33320879
Sus scrofa	wild boar	33320893
Sus scrofa	wild boar	33320907
Sus scrofa	wild boar	33320921
Sus scrofa	wild boar	33320935
Sus scrofa	wild boar	33320949
Sus scrofa	wild boar	37962869
Sus scrofa	wild boar	45826169
Sus scrofa	wild boar	45826183
Sus scrofa	wild boar	45826197
Sus scrofa	wild boar	45826211
Sus scrofa	wild boar	4958951
Sus scrofa	wild boar	5835862
Sus scrofa	wild boar	83779131
Sus scrofa	wild boar	83779145
Sus scrofa	wild boar	84181679
Sus scrofa	wild boar	90969035
Sus scrofa	wild boar	94981320
Sus scrofa	wild boar	95116704
Tachyglossus aculeatus	short-beaked echidna	18077882
Talpa europaea	European mole	7212513
Tamandua tetradactyla	southern tamandua	21449946
Tarsipes rostratus	honey possum	56548719
Tarsius bancanus	western tarsier	14582815
Tarsius bancanus	western tarsier	14602225
Thryonomys swinderianus	greater cane rat	12667705
Thylamys elegans	elegant fat-tailed opossum	45685705
Trachypithecus obscurus	spectacled langur	60392086
Trachypithecus obscurus	spectacled langur	62161253
Trichosurus vulpecula	silver-gray brushtail possum	13752501
Trichosurus vulpecula	silver-gray brushtail possum	15079189
Tupaia belangeri	northern tree shrew	9997000
Urotrichus talpoides	Japanese shrew mole	32469179
Urotrichus talpoides	Japanese shrew mole	32526828
Ursus americanus	American black bear	19110544
Ursus americanus	American black bear	19343488
Ursus arctos	grizzly bear	19110558
Ursus arctos	grizzly bear	19343502
Ursus maritimus	white bear	19110572
Ursus maritimus	white bear	19343516
Vombatus ursinus	common wombat	18077896
Zaglossus bruijni	long-beaked echidna	54112085

Sturnira lilium dataset

Species name	Common name	GenBankID COI	GenBankID *cytb*
Sturnira lilium	Little Yellow-shouldered Bat	EF546846	EF536933
Sturnira lilium	Little Yellow-shouldered Bat	EF546845	EF536934
Sturnira lilium	Little Yellow-shouldered Bat	EF546844	EF536935
Sturnira lilium	Little Yellow-shouldered Bat	EF546843	EF536936
Sturnira lilium	Little Yellow-shouldered Bat	EF546842	EF536937
Sturnira lilium	Little Yellow-shouldered Bat	EF546841	EF536938
Sturnira lilium	Little Yellow-shouldered Bat	EF546840	EF536939
Sturnira lilium	Little Yellow-shouldered Bat	EF080685	EF536940
Sturnira lilium	Little Yellow-shouldered Bat	EF080686	EF536941
Sturnira lilium	Little Yellow-shouldered Bat	EF080687	EF536942
Sturnira lilium	Little Yellow-shouldered Bat	EF080688	EF536943
Sturnira lilium	Little Yellow-shouldered Bat	EF546839	EF536944
Sturnira lilium	Little Yellow-shouldered Bat	EF546818	EF536945
Sturnira lilium	Little Yellow-shouldered Bat	EF546838	EF536946
Sturnira lilium	Little Yellow-shouldered Bat	EF546837	EF536947
Sturnira lilium	Little Yellow-shouldered Bat	EF546836	EF536948
Sturnira lilium	Little Yellow-shouldered Bat	EF546835	EF536949
Sturnira lilium	Little Yellow-shouldered Bat	EF546834	EF536950
Sturnira lilium	Little Yellow-shouldered Bat	EF546833	EF536951
Sturnira lilium	Little Yellow-shouldered Bat	EF546832	EF536952
Sturnira lilium	Little Yellow-shouldered Bat	EF546831	EF536953
Sturnira lilium	Little Yellow-shouldered Bat	EF546830	EF536954
Sturnira lilium	Little Yellow-shouldered Bat	EF546829	EF536955
Sturnira lilium	Little Yellow-shouldered Bat	EF546828	EF536956
Sturnira lilium	Little Yellow-shouldered Bat	EF546827	EF536957
Sturnira lilium	Little Yellow-shouldered Bat	EF546826	EF536958
Sturnira lilium	Little Yellow-shouldered Bat	EF546825	EF536959
Sturnira lilium	Little Yellow-shouldered Bat	EF546824	EF536960
Sturnira lilium	Little Yellow-shouldered Bat	EF546823	EF536961
Sturnira lilium	Little Yellow-shouldered Bat	EF546822	EF536962
Sturnira lilium	Little Yellow-shouldered Bat	EF546821	EF536963
Sturnira lilium	Little Yellow-shouldered Bat	EF546820	EF536964
Sturnira lilium	Little Yellow-shouldered Bat	EF546819	EF536965
Sturnira lilium	Little Yellow-shouldered Bat	EF080689	EF536966

Bat dataset

GenBank ID for COI barcodes: EF079971– EF080810
